# A comprehensive scoping review to identify standards for the development of health information resources on the internet

**DOI:** 10.1371/journal.pone.0218342

**Published:** 2019-06-20

**Authors:** Noha Abdel-Wahab, Devesh Rai, Harish Siddhanamatha, Abhinav Dodeja, Maria E. Suarez-Almazor, Maria A. Lopez-Olivo

**Affiliations:** 1 Section of Rheumatology and Clinical Immunology, Department of General Internal Medicine, The University of Texas MD Anderson Cancer Center, Houston, Texas, United States of America; 2 Rheumatology and Rehabilitation Department, Faculty of Medicine, Assiut University Hospitals, Assiut, Egypt; King Abdulaziz University, SAUDI ARABIA

## Abstract

**Background:**

Online health information, if evidence-based and unbiased, can improve patients’ and caregivers’ health knowledge and assist them in disease management and health care decision-making.

**Objective:**

To identify standards for the development of health information resources on the internet for patients.

**Methods:**

We searched in MEDLINE, CINAHL, Scopus, Web of Science, and Google Scholar for publications describing evaluation instruments for websites providing health information. Eligible instruments were identified by three independent reviewers and disagreements resolved by consensus. Items reported were extracted and categorized into seven domains (accuracy, completeness and comprehensiveness, technical elements, design and aesthetics, usability, accessibility, and readability) that were previously thought to be a minimum requirement for websites.

**Results:**

One hundred eleven articles met inclusion criteria, reporting 92 evaluation instruments (1609 items). We found 74 unique items that we grouped into the seven domains. For the accuracy domain, one item evaluated information provided in concordance with current guidelines. For completeness and comprehensiveness, 18 items described the disease with respect to various topics such as etiology or therapy, among others. For technical elements, 27 items evaluated disclosure of authorship, sponsorship, affiliation, editorial process, feedback process, privacy, and data protection. For design and aesthetics, 10 items evaluated consistent layout and relevant graphics and images. For usability, 10 items evaluated ease of navigation and functionality of internal search engines. For accessibility, five items evaluated the availability of websites to people with audiovisual disabilities. For readability, three items evaluated conversational writing style and use of a readability tool to determine the reading level of the text.

**Conclusion:**

We identified standards for the development of online patient health information. This proposed instrument can serve as a guideline to develop and improve how health information is presented on the internet.

## Introduction

The internet has become the new “first aid,” often the first place for patients to get health information given its ease of access. Health information on the internet has rapidly grown over the past two decades, from 10,000 websites providing health care information in 1997 to millions of websites at present [1, 2]. According to a national survey done in 2013 in the United States by the Pew Research Center, one in three American adults have used the internet to find information about a medical condition [3]. Patients not only browse the internet for health information before consulting with a physician [4] but also expect doctors to recommend useful resources and supplementary quality information services [5]. Although health information provided on the internet cannot replace the advice of a health care professional, it can serve as a readily available source of information, which, if evidence-based and unbiased, can improve patients’ knowledge [6, 7].

Websites providing health information for patients should follow high quality standards and be comprehensible. According to a study performed by the US National Center for Education Statistics, nine of ten Americans have difficulty understanding health information on the internet [8]. Multiple instruments have been developed to help health providers identify websites with reliable health information that can be recommended to their patients. In 2002, Eysenbach et al [9] performed a systematic review to establish a methodological framework on how the quality of health information on the internet should be evaluated. The authors identified seven domains that were thought to be a minimum requirement: (i) accuracy, (ii) completeness and comprehensiveness, (iii) technical elements, (iv) readability, (v) design and aesthetics, (vi) accessibility, and (vii) usability. However, no items were provided for each domain for detailed evaluation. These domains were further divided by Zhang et al in 2015 [10]. The technical elements were separated into currency of the information provided, credibility, and privacy and data protection. In addition, usability was separated into navigability, interactivity, and cultural sensitivity. Although items to evaluate these criteria were presented, no explanatory definitions for each item were provided.

To date, no tools have been compiled for reporting items and providing explanatory definitions of the original seven domains identified by Eysenbach et al. We have conducted a review of available instruments used to evaluate websites providing health information for patients to identify relevant items to consider for the development of online health information resources.

## Materials and methods

We report our methods and results according to the Preferred Reporting Items for Systematic Review and Meta-Analysis statement (S1 Table).

### Eligibility criteria

We included articles describing evaluation instruments that were previously used to evaluate the quality of websites. We excluded articles that provided instruments for librarians, software engineers, or universities, as well as those providing items for advertising or measuring consumer perceptions. We also excluded articles that did not provide items while reporting domains similar to those described by Eysenbach et al [9].

### Information sources

We performed a literature search in MEDLINE, CINAHL, Scopus, and Web of Science databases, as well as Google Scholar.

### Search

Search terms included “online,” “Internet,” “information,” “resource,” “evaluate,” “assess,” “tool,” “instrument,” “questionnaire,” “website,” “web-site,” and “web site.” The search was performed for published articles, unpublished articles, and online instruments irrespective of language and region. References of the retrieved articles were also searched manually for original citations and additional instruments otherwise not found.

### Study selection

Three reviewers (DR, NA-W, and HS) independently selected studies reporting instruments to evaluate health care or general websites. Agreement was achieved through consensus.

### Data collection process and data items

Two reviewers (DR, HS) independently extracted all items from the retrieved instruments. Articles in a language other than English (Korean, Spanish, and French) were translated by collaborators proficient in the original language, or by Google translator, to retrieve the items. We included all items that were used to assess at least one of the domains proposed by Eysenbach et al. We did not consider items that evaluated metadata standards, html coding of websites, or financial transactions.

### Synthesis of results

Each item was considered relevant if the reviewers reached a consensus to include it in the proposed instrument, with final selection achieved through third party adjudication if needed. Items describing similar criteria were grouped together under the Eysenbach et al proposed domains, and the frequency of each item in the original retrieved instrument was calculated.

## Results

### Study selection

One hundred sixty articles were identified through databases and 80 through hand searching. After duplicate removal, the full texts of 239 unique articles were assessed for eligibility. One hundred eleven articles met our inclusion criteria, providing 92 distinct evaluation instruments (S1 Fig).

### Study characteristics

The characteristics of the 92 included instruments are described in Table 1. The creator, validation, and domains and items proposed by each individual instrument are detailed in S2 Table. We found that 64 instruments (70%) were developed by the authors and 28 (30%) by various organizations. Eighty-three instruments (90%) evaluated health care websites or websites in general (e.g., the DISCERN tool evaluated reliable health information and quality of treatment options[11]), and nine (10%) evaluated disease-specific websites (e.g., measurable criteria for credibility score for diabetes websites [12]). Ninety instruments (98%) targeted the general population, and two (2%) were specific for either low-literacy or elderly populations. Eighty-three instruments (90%) included items for more than one domain of interest and the remaining for only a specific domain. Only 28 instruments (30%) had been validated by independent reviewers and measured interrater reliability. However, none of the identified instruments covered all domains.

**Table 1:** Characteristics of the included instruments (n = 92).

Characteristic	No. of instruments (%)
Creator/developer	
Authors	64 (70)
Organizations	28 (30)
.org	14
.com	1
.edu	5
.gov	5
.eu	3
Target website	
Health care-specific websites or websites in general^a^	83 (90)
Disease-specific	9 (10)
Alzheimer disease	1
Neuropathology	1
Female urinary incontinence	1
Multiple sclerosis evaluation instrument	1
Diabetes	1
Breastfeeding	1
Hypoactive sexual desire disorder	1
Vaccination	1
Bipolar disorder	1
Target audience	
General population	90 (98)
Low literacy	1 (1)
Elderly	1 (1)
Domain of interest	
More than one domain or item	83 (90)
Usability	3 (3)
Accessibility	3 (3)
Technical elements (privacy)	2 (2)
Design of interface	1 (1)
Instrument validation	
Not validated	56 (61)
Validated	28 (30)
Not reported	8 (9)

^a^Nine of these instruments did not not have any specific name. In S2 Table, we refer to them by the author’s name.

### Synthesis of results

A total of 1,609 items were retrieved from the included instruments (See S2 Table). After removing duplicates, we categorized 74 unique items into the domains proposed by Eysenbach et al. Agreement on item suitability for each domain was achieved by consensus. The final items are listed in Table 2, along with the frequency of reporting of each item in the original 92 instruments retrieved from the literature search.

**Table 2:** Frequency with which each item was reported in the 92 instruments identified in the literature search.

Item No.	Item and domain	No. (%) of instruments in the literature that included the item
	Accuracy	
1	Based on guidelines/standards of care, current research using MEDLINE searches, textbooks, expert consultation or author opinion	77 (84)
	Completeness and comprehensiveness	
1	Definition	9 (10)
2	Epidemiology	5 (5)
3	Etiology, with extent of impact of each contributing factor	6 (7)
4	Pathogenesis with simple explanation	5 (5)
5	Clinical features, including signs and symptoms	11 (12)
6	Diagnosis (laboratory and/or radiologic)	7 (8)
7	Management, with description of each treatment (including prophylactic and screening) and how it works, mechanism, and contraindications	17 (18)
8	Self-management	9 (10)
9	Balance between benefits and harms of treatment	18 (20)
10	Costs of treatment	4 (4)
11	Monitoring	6 (7)
12	Complications	10 (11)
13	Consequences of no adherence	5 (5)
14	Areas of uncertainty	3 (3)
15	Questions to discuss with those involved in the patient’s care	8 (9)
16	Interaction (problems or questions presented for response such as quizzes) or use of interactivity (medical evaluation with information provided by the user, discussion groups, forums, consultation, or chat window)	25 (27)
17	Cases or examples of desired behavior (e.g., user testimonials)	8 (9)
18	Subdivision of complex topics (so that readers can experience small successes in understanding)	9 (10)
	Technical elements	
1	Disclosure of authorship	55 (60)
2	Disclosure of author affiliation	27 (29)
3	Disclosure of author credentials (name and credentials of all human or institutional providers of information, including dates when credentials were received)	39 (42)
4	Disclosure of physician credentials	10 (11)
5	Author is a recognized authority (not provided by health professionals or specialists)	27 (29)
6	Disclosure of ownership (who is responsible for the website)	36 (39)
7	Disclosure of sponsorship (all sources of funding for website grants, sponsors, advertisers, and nonprofit or voluntary assistance), preferably in homepage or as a link in the about page	47 (51)
8	Editorial review process (clear statement describing procedure for selecting content and disclosure of team members and peer review)	19 (21)
9	Clear hierarchy of evidence (expression of opinion as opposed to relevant information)	4 (4)
10	Advertisements distinctly labeled and separated from website content; only one advertisement per screen and none on homepage	23 (25)
11	Statement of purpose (statement clearly declaring that the information on the website is not meant to replace the advice of a health professional; objectives and aims clearly described)	29 (32)
12	General disclosures (educational, nonprofit, or commercial; URL affiliation of edu, org, com, net, gov, or mil)	43 (47)
13	Identification of target audience (further details on purpose of website; multiple audiences could be defined at different levels)	31 (34)
14	Clear sources (clear statement of sources for all information provided, including date of publication of source)	40 (43)
15	References (benefits or performance of any medical or surgical treatment, commercial product, or service; all claims backed up with scientific evidence from medical journals, reports, or others; all brand names identified; unless the purpose of the website is clearly stated to be the commercial platform of a particular product, alternative therapies or products, including generics, are mentioned)	45 (49)
16	Creative commons license, if material is copyrighted	11 (12)
17	General disclaimers (privacy and data protection policy; system for the processing of personal data, including processes invisible to users, clearly defined in accordance with community data protection legislation)	23 (25)
18	Information on data collection and who can access it, how it is accessed (including statistics)	16 (17)
19	Option to opt in/out of a subscription service (newsletter, updates)	5 (5)
20	Message alert if cookies are used (with option to disable)	5 (5)
21	Message alert when leaving a secured website	5 (5)
22	Date of content creation	58 (63)
23	Date of the last update (clear and regular updating of the website, with date of update clearly displayed for each page and/or item as relevant; regular checking of relevance of information)	34 (37)
24	Date of planned technical maintenance (announced ahead of time)	5 (5)
25	Links (all efforts made to ensure that partnering or linking to other websites is undertaken only with trustworthy individuals and organizations that comply with relevant codes of good practice; no broken links; distinct difference between non-visited and visited links)	52 (57)
26	Contact information (phone and fax numbers for customer service, email address of author and editorial team, and location of headquarters, including street address and country)	76 (83)
27	Feedback mechanisms (user feedback and appropriate oversight responsibility, such as a named quality compliance officer for each site); response time for feedback; feature to rate the usefulness of information and educational impact on users	43 (47)
	Design and aesthetics	
1	Visual aspect of the website (all material; alignment, scroll bars, etc)	22 (24)
2	Quality of visual presentation (also whether website can be viewed from a partial window/restore down option)	14 (15)
3	Menu (directional icons, bars, indicators, listing, indexes)	29 (32)
4	Typography (text and headlines in uppercase and lowercase; type size at least 12 points; use of bold type, color, and different sizes; ability to change the size and font)	24 (26)
5	Appropriate grammar (also, abbreviations and acronyms spelled out at first use on each page; jargon defined or glossary page included)	23 (25)
6	Consistent layout (illustrations adjacent to the related text; layout and sequence of information consistent so that flow of information is predictable; visual cueing devices such as boxes, arrows, and shading used to direct attention to key content; pages do not appear cluttered; use of color supports and does not distracting from the message; line lengths of 30 to 50 characters and spaces; sentences with 20 or fewer words; paragraphs with five or fewer sentences)	34 (37)
7	Subheadings and chunking (lists longer than three to five items partitioned into small chunks, lists grouped under descriptive subheadings)	17 (18)
8	Relevant graphics and images (present key messages visually so the reader can grasp key ideas from the illustrations alone, graphics explained)	35 (38)
9	Appropriate type of material (text, graphics, tables, equations, audio, and video), cover images (friendly, attract attention, clearly portray the purpose of the materials), illustrations (line drawings likely to be familiar to readers), and media (no autoplay, minimal use of animation)	26 (28)
10	Browser compatibility	10 (11)
	Usability	
1	Easy navigation (well organized, index, table of contents, icons for navigation, easiness to return to the previous page, home page redirection to unintended websites, site map, help function, frequently asked questions, breadcrumbs, show previous and next topics)	46 (50)
2	Internal search engine (with instructions to use)	39 (42)
3	Functionality (supports content, e.g., calculations)	3 (3)
4	Registration and password protection for restricted content limited to three screens with cues	15 (16)
5	Option to download/print materials	18 (20)
6	Large files include space for the size	5 (5)
7	Graphic files with “mouse over” indication of graphical content	6 (7)
8	Speed (page takes less than 5 seconds to load, including text and images, at less than 50 kilobits per second bandwidth)	17 (18)
9	Dublin core tags (content is tagged)	3 (3)
10	Page does not require other computer applications for viewing (e.g., Adobe Acrobat, Microsoft PowerPoint) or links are provided when necessary to download needed browser plug-ins	11 (12)
	Accessibility	
1	Compliance with World Wide Web Consortium 2018 guidelines (website is available to people with disabilities or low-end technology)	26 (28)
2	Findability (easiness to find content, depth of navigation, how many clicks or webpages before a particular topic is reached)	20 (22)
3	Appropriate color contrast (font and background color are contrasting but are reader-friendly to avoid eye strain)	24 (26)
4	Cultural match: appropriate language (preferred language based on target audience, e.g., English. When the content is translated from one language to another, cultural match is important because some words may have different meanings in various cultures and languages)	26 (28)
5	Cultural match: images and examples presented in realistic and positive ways	4 (4)
	Readability	
1	Use of a tool (e.g., Flesch-Kincaid Grade Level Index; SMOG Readability Grading; Fry Readability Graph; Gunning Fog Index)	17 (18)
2	Writing style (conversational style, active voice, simple sentences)	23 (25)
3	Sentence construction (consistently provides context before presenting new information)	17 (18)

Interpretations of each identified readability tool are summarized in Table 3. In the following subsections, we describe in detail the items we included under each domain.

**Table 3:** Readability tools and their interpretations.

Readability tool	Interpretation
Sherman [13]	Reduce the length of each sentence to 20 words; written language becomes more efficient by becoming like spoken language
Thorndike [14]	Higher frequency of a word appearing in a specific list of 10,000 words indicates higher readability; in an updated version, the list of words increased to 30,000
Lively and Pressey measuring method [15]	Statistical analysis to grade the reading difficulty of textbooks using the Thorndike list
Patty and Painter [16]	Combined use of Thorndike list and vocabulary diversity to calculate frequency and vocabulary burden of textbooks
Lorge index [17]	Takes into account the average length of sentences, number of prepositional words, and number of hard words not in the Dale list; tool was suggested to be useful for adults as well as children
Dale-Chall [18, 19]	Predicts grade level of a text using 100 words sampled from the text and a relative number of words not on the Dale list of 3,000 words
Flesch reading ease formula [20]	Reports “human interest” score from zero (no human interest) to 100 (full of human interest)
Spache [21]	Reports the grade level of the text, recommends scoring at least three samples and calculating the average for a reliable estimate of reading difficulty
Powers-Sumner-Kearl [22]	Years of schooling (grade level) needed to read and understand the text
Bormouth [23]	Years of schooling (grade level) needed to read and understand the text
Automated readability index [24]	Years of schooling (grade level) needed to read and understand the text
Fry readability graph [25]	Years of schooling (grade level) needed to read and understand the text
Gunning Fog index [26]	Years of schooling (grade level) needed to read and understand the text
The Simple Measure of Gobbledygook (SMOG) grading [27]	Years of schooling (grade level) needed to read and understand the text
Flesch-Kincaid grade level [28]	Years of schooling (grade level) needed to read and understand the text
Coleman [29]	Years of schooling (grade level) needed to read and understand the text
Raygor estimates graph [30]	Years of schooling (grade level) needed to read and understand the text
FORCAST readability [31]	Years of schooling (grade level) needed to read and understand the text

### Accuracy

Accuracy, also often referred to as reliability, is defined as the extent to which the information provided on the website is in concordance with current standards [32]. Items found for this domain were combined into one because all were measuring the same concept: information should be based on current guidelines or standards of care. That is, the information provided should be a summary of current evidence according to clinical practices guidelines, textbooks, and/or expert consultation when there is no evidence about the topic.

### Completeness and comprehensiveness

Eysenbach et al previously proposed completeness and comprehensiveness as one domain. For completeness, also sometimes called coverage, or scope, any website providing health information should cover the main concepts of the topic. We judged that the following eighteen items should ideally be covered to improve understanding of the condition of interest. The disease should be accurately defined (item 1), and the epidemiology, etiology, pathogenesis, clinical features, method of diagnosis, standard management, and typical self-management of the disease (items 2–8) should be reported. In addition, the beneficial and harmful effects of each treatment (item 9) and treatment costs (item 10) should be explicitly mentioned. Disease monitoring (item 11), complications (item 12), and consequences that could ensue if no treatment is used (item 13) should also be described in detail. Areas of uncertainty (item 14) and questions to be discussed with those involved in the patient's care (item 15) should also be mentioned. For comprehensiveness, a platform for users to interact (item 16) should be included, or cases and/or examples of desired behavior (item 17) should be modeled or shown. Lastly, complex topics should be subdivided so that readers can experience small successes in understanding (item 18).

### Technical elements

According to Eysenbach et al [9], technical elements depict the way in which information is presented to users. This domain evaluates the trustworthiness of content and the confidentiality of any personal data collected. Twenty-nine items were included under technical elements; 24 of them were reported by Eysenbach as the most frequently used [33]. First, authorship (item 1), author affiliations and credentials (items 2 and 3), physician credentials (item 4), ownership (item 6), and sponsorship (item 7) should be disclosed. If the author is a recognized authority, this should be mentioned (item 5). The editorial review process (item 8) should be described. The hierarchy of evidence (item 9) should also be clear, and advertisements on the website should be distinctly labeled as such and limited to one per screen, with none on the homepage (item 10). The objective of the websites should be clear; a statement explicitly declaring that the information on the websites is not meant to replace the advice of health professionals and clearly describing the aim of the website should be included on the homepage (item 11)[34]. The type of website should also be described as a general disclosure (item 12)—i.e., whether the website serves an educational, nonprofit, or commercial purpose. The website should clearly define its target audience (item 13) and describe sources of information (item 14) and provide references for its claims (item 15). When the content is copyrighted, a creative commons license should be provided (item 16).

The website should also provide detailed disclaimers covering the privacy and data protection policies (item 17), abiding by the directives given by the National Research Council Committee on Maintaining Privacy Security [35] and the European Commission [36]. Data collection procedures (item 18), ability to opt in and out of subscriptions (item 19), and usage of cookies (item 20) should be explicitly mentioned. The website should display an alert when the user is leaving a secured page (item 21). The date of content creation (item 22) should be provided, along with the date of update for each page (item 23), and the date of any technical maintenance should be disclosed beforehand (item 24). Third party links that abide by ethical principles should be provided (item 25). Contact information (item 26) and a feedback mechanism (item 27) should also be provided.

### Design and aesthetics

Design and aesthetic elements are the first thing to catch the attention of visitors to a website and can facilitate understanding, the speed with which website visitors can find what they are looking for, and their belief that the website is trustworthy [37]. Ten items were included for this domain. Pleasant visual presentation (item 1) is key, with an option to view the website in a partial window or restore down (item 2) and distinct menus with directional icons, bars, indicators, listings, and indexes (item 3). Text should be at least 12 points with appropriate use of fonts, colors, and capitalization, and users should have the option to change the type size or font (item 4). Appropriate grammar should be used, abbreviations and acronyms should be spelled out the first time they are mentioned on each page, and the use of medical jargon should be limited and clearly defined on a glossary page (item 5). The layout should be consistent (item 6) and the sequence of information should be clear throughout the website, with bigger topics subdivided into subheadings and short lists (item 7). Because images present key messages visually to the reader, relevant images and graphics with an explanation should be provided (item 8). Cover images on the website should be friendly, media could be used to communicate with users, and autoplay should be disabled by default if any audio or video is present (item 9). The website should be compatible with all browsers (item 10).

### Usability

Usability is defined as “the capability to be used by humans easily and efficiently” [38]. Usability is critical because information is effective only if it can be used with ease. Ten items were included in the usability domain after we combined items measuring the same concept. Content should be well organized ideally with an index, table of contents, navigation icons, breadcrumbs, ability to easily return to the previous and proceed to next pages, site map, and help function favoring an optimal navigation experience (item 1). An internal search engine (item 2) and functionality supporting content such as calculators (item 3) are recommended. If any registration and password protection is present, registration should be done within three screens (item 4). All content should be in a printer-friendly format with an option to download (item 5), and if the content requires large file sizes, the website should mention the size of the file along with the estimated time to download (item 6). Graphics files should be marked with a “mouse over” distinctly indicating the presence of graphical content (item 7). The loading time of the webpage should perform according to standards even at relatively low bandwidth speeds; the current average rate is 512 kilobits per second (this may change with time; item 8). The website content should be tagged with Dublin core tags [39], a component of metadata that increases the searchable index of the website content (item 9). The page should generally not require additional computer applications, and if additional applications are needed, working links for application download should be provided (item 10).

### Accessibility

Accessibility is defined by the World Wide Web Consortium (W3C) as “making content more accessible to people with the wider range of disabilities including blindness and low vision, deafness, speech disabilities, learning disabilities, cognitive limitations, limited movement, photosensitivity, and combinations of these” [40]. The W3C proposed Web Content Accessibility Guidelines, which stated ways to make content on the internet accessible to people with various degrees of disabilities [40]. W3C recommended providing an option of increasing the type size and changing the font and background color, as well as offering podcasts for voice reading and text and making all content accessible by keyboard, among other recommendations.

Using the W3C guidelines as a basis, we included five items in accessibility. The website should comply with the W3C 2018 guidelines (item 1). Easy findability (i.e., the ability to search content using minimal steps; item 2) and appropriate color contrast (item 3) should also be present. Comprehension has been found to be better when an individual is addressed in their native language. Thus, the language used on the website should be based on the target audience (item 4), and when the content is translated from one language to another, cultural match is important because some words may have different meanings in various cultures and languages. Sign language interpretation for all recorded materials (audio or video) should be provided when relevant to the target audience. The translated content should be proofread by a language expert before it is released on the website. Similarly, the cultural match of images and examples should be taken into account (item 5).

### Readability

Readability is the ease with which text can be read [41]. We considered three items from our review: determination of the content level (item 1), appropriate writing style (item 2), and sentence construction (item 3). The US National Institutes of Health and the American Medical Association recommend that readability scores for content written on a website range from sixth- to eighth-grade level [42, 43]. Among the 92 instruments identified, there were 18 readability tools used to determine the years of education needed to understand the content (Table 3). The most commonly used were the Simple Measure of Gobbledygook grading [27], Gunning Fog index [26], and Flesch-Kincaid grade level [20, 28]. It is important to use a readability tool only to determine the reading level of the text and not for assessing the overall suitability of online material, because readability tools fail to take into account comprehension and the role of the reader [44, 45]. Regarding the writing style and the sentence construction, information on the website should be conveyed in an active voice in small sentences and complex information should be broken down.

## Discussion

Although the internet has the capacity to disseminate rich medical information, it can also disseminate inaccurate, biased, and out-of-date information, which can have adverse effects on consumers of health information (e.g., patients and caregivers) [2]. The content of the website has been found to be as important as the design to gain the trust of the viewers and to easily disseminate health-related information if the information is provided in an interactive manner [37, 46]. Therefore, an instrument that incorporates the seven fundamental domains to provide online health information to the patients, which is likely to be different from that provided by print media, is needed [2].

Such an instrument is long overdue. Most studies that previously evaluated the quality of information on websites used criteria derived by personal opinion. The purpose of our study was to identify standards for the development of health information resources on the Internet for patients. In our review, we incorporated all of the domains suggested by Eysenbach et al and Zhang et al [9, 10]. However, one of these studies did not provide the items that should be considered for each domain for in-depth evaluation and the other did not define the items presented. We reviewed the literature and retrieved 92 evaluation instruments. We then defined and summarized all of the items proposed by previous studies and included the retrieved items under their respective domains. We have thus proposed an instrument that furthers the contributions of Eysenbach et al and Zhang et al to the medical literature, incorporating detailed guidelines for the development of websites providing health information to patients.

In 1995, the Economic and Social Council of the United Nations set up the Health on the Net foundation guidelines for online health information. These guidelines included components of completeness and technical criteria but did not include accuracy, readability, design, usability, and accessibility [34]. In the same year, the *Journal of the American Medical Association* published benchmark criteria to evaluate websites on authorship, attribution, disclosure, and relevance (i.e., whether the information was up to date) but did not cover accuracy, readability, usability, and accessibility [2]. Thereafter, the DISCERN instrument was developed, and this evaluated the reliability and quality of information on treatment choices, including accuracy, completeness, and technical criteria [11]. The principles governing American Medical Association websites were the first to offer additional guidelines for advertising, sponsorship, privacy, confidentiality, and principles for e-commerce, but these guidelines also did not mention readability, usability, and accessibility [47]. Recently, the US Agency for Healthcare Research and Quality created two patient education materials assessment instruments for assessing the understandability and accountability of print and audiovisual patient education materials. These instruments evaluated content, readability, organization, and design, but they did not mention accessibility and usability [48]. All of these guidelines focused only on specific domains for evaluation, and none of them mentioned usability or accessibility. These domains are important and should be considered because the information on the internet can be beneficial only if it is easily conveyed to the audience. Evidence suggests that the navigation experience (e.g., website layout, accessibility) is associated with the trust that users place in the information provided. When information is better displayed and easy to find, users pay more attention, which may result in increased readability, reliability, and improved learning [49].

Several other systematic reviews have evaluated the rating instruments that are commonly used for evaluation of the health-related information on the internet [9, 50–54]. Kim et al developed an instrument for evaluation of health-related websites using 12 categories grouping all criteria they identified from the literature search, but that instrument did not include evaluation of readability [53]. All others did not propose any instruments and used only limited categories that were previously proposed for evaluation and were missing evaluation of important domains such as usability and accessibility [50–52] (see S3 Table). These authors were contacted to determine their current activities to further refine their published work and avoid duplication of efforts, but such a comperhensive instrument is not under development.

Because the internet has grown at a fast pace and personal health information is being shared among all platforms, we have taken into account all aspects of privacy recommended by authorities, including the US National Research Council proposed guidelines for protecting electronic health information and preventing inappropriate sharing of the personal information of website users [35], as well as the ePrivacy Directive [36] (an updated set of criteria derived from the European Commission devised eEUROPE 2002). These are a set of quality criteria for health-related websites that provide directives for transparency and honesty, authority, privacy and data protection, updating of information, accountability, and accessibility [55].

Any content provided to patients can be beneficial only if the patient will be able to comprehend the information, and according to the US National Center for Education Statistics Survey, 50% of Americans are unable to understand above an eighth grade reading level [8]. The Suitability Assessment of Material guidelines proposed ways to make content understandable for a patient with low literacy skills. These guidelines also pointed to the cultural relevance of language, images, typography and layout, and multimedia materials for patients [56]. We advocate using a readability tool in addition to other items related to the writing style and sentence construction, as recommended by the Suitability Assessment of Material guidelines [56].

Our proposed instrument contains extensive and precise elements based on, to the best of our knowledge, the available guidelines. The instrument includes all necessary domains and proposes a detailed definition of items, which will pave the way for efficient development of websites providing health care information to patients. Our instrument is further strengthened by the inclusion of the Web and Usability Guidelines [57], the Guide to Writing and Designing Easy-to-Use Health Websites [57], the Accessible Health Information Technology Guidelines for Populations with Limited Literacy [55, 58], and the Web Content Accessibility Guidelines [40]. Moreover, our instrument is not restricted to the development of disease-specific content, although it can be used to develop websites with a specific focus. Although we sought to generate a comprehensive list of all items required for our instrument, we did emphasize some disease-specific components, such as pathogenesis and coverage of areas of uncertainty, as well as website-specific components such as internal search engines, breadcrumbs, and Dublin core tags, which were mentioned in a few studies. The items included in our instrument will serve as standards for developing future online health information. We included an extensive list of domains and items in an attempt to ensure comprehensiveness, however, the Internet is constantly changing and new technologies may dictate future modifications to the number and complexity of the items included. All collected information is available as a supplementary material.

## Conclusion

In conclusion, as the reach of the internet grows and evolves, the components needed for the development of online health information must be continually improved, and new components must be added. A static instrument will not always suffice to report information over such a dynamic platform as the internet. Therefore, development criteria need to be periodically revised with the addition of new aspects or modification of existing aspects while retaining those considered fundamental [47].

## Supporting information

S1 Fig Study selection flowchart. (DOCX)

S1 Table PRISMA checklist. (DOCX)

S2 Table Instruments included in our analysis (n = 92). (DOCX)

S3 Table Summary of previous systematic reviews evaluating the rating instruments used for evaluation of health-related information on the internet. (DOCX)
