# Supplementary material for: A comprehensive scoping review to identify standards for the development of health information resources on the internet
Source: PLoS One. 2019 Jun 20;14(6):e0218342. doi: 10.1371/journal.pone.0218342 (PMC6586310; doi:10.1371/journal.pone.0218342)
Supplement: S1 Fig — (DOCX) [file pone.0218342.s001.docx]

**S1 Figure.** Study selection flowchart

**Articles identified through databases**

**(n =160)**

**Articles identified through bibliography hand searching**

**(n =80)**

**Unique articles after duplicates removal
(n = 239)**

**Full-text articles excluded** **(n =128)**

1. No guidelines are reported (n =80)
2. Guidelines for librarians, software engineers, universities and healthcare professionals (n =27)
3. Guidelines for measuring consumer perceptions (n =10)
4. Guidelines for advertising (e-commerce and marketing, drug advertising, information system) (n =7)
5. No items are provided, while reporting similar domains to Eysenbach (n=3)
6. Irretrievable^a^ (n =1)

**Full-text articles assessed for eligibility (239)**

**(n = 201)**

**Articles included (111)**

**Tools (n =92)**

^a^Irretrievable: Exhausted all possible resources and library was not able to retrieve a copy.
