# Supplementary material for: A comprehensive scoping review to identify standards for the development of health information resources on the internet
Source: PLoS One. 2019 Jun 20;14(6):e0218342. doi: 10.1371/journal.pone.0218342 (PMC6586310; doi:10.1371/journal.pone.0218342)
Supplement: S2 Table — (DOCX) [file pone.0218342.s003.docx]

**S2 Table:** Instruments included in our analysis (n = 92).

| **Instrument name** | **Creator of instrument** | **Valid** | **Domain** | **Items** |
| --- | --- | --- | --- | --- |
| 1. Accessible Health Information Technology for Populations with Limited Literacy [1] | Authors for  Agency for Healthcare Research and Quality,  US Department of Health and Human Services | NR | 1. Accessibility | 1. Cultural match, appropriate language for the region. 2. Appropriate grammar. 3. Appropriate writing style. 4. Appropriate sentence construction. 5. Use of a readability tool. 6. Target audience clearly defined. 7. Relevant graphics and images. 8. Subheadings and chunking. 9. Appropriate color contrast. 10. Layout with appropriate illustrations adjacent to the related text; visual cueing devices such as boxes, arrows, and shading used to direct attention to key content. 11. Visual aspect. 12. Appropriate typography. 13. Menu (directional icons, bars, indicators, listing, indexes). 14. Feedback mechanisms, response time for feedback, feature to rate the usefulness of information. 15. Cultural match: images and examples presented in realistic and positive ways. 16. Browser compatibility. 17. Easy navigation. 18. Quality of visual presentation. 19. Internal search engine. 20. Links provided with responsible partnering. 21. Option to download/print materials. 22. Available to people with disabilities or low-end technology. 23. Email address. |
| 2. Automated Analysis of Web Pages [2] | Authors | No |  | 1. Disclosure of authorship. 2. Links provided with responsible partnering. 3. Disclosure of date of creation. 4. Disclosure of sponsorship. |
| 3. Certified Medical Web Online instrument^a^ [3] | Medical Association of Barcelona | No | 1. Identification 2. Content   (accuracy)   1. Confidentiality 2. Security 3. Advertising and funding 4. Virtual consultation 5. Compliance with code of ethics | 1. Email address. 2. Disclosure of ownership. 3. Disclosure of author affiliation. 4. Country. 5. General disclosures (educational, nonprofit, or commercial). 6. Disclosure of authorship. 7. Disclosure of date of last update. 8. Menu (directional icons, bars, indicators, listing, indexes). 9. Links provided with responsible partnering. 10. References. 11. Guidelines/standards of care. 12. Based on current research using MEDLINE searches. 13. Expert consultation or personal opinion of the author. 14. Cultural match: appropriate language for the region. 15. Data collection, who can access it, how it is accessed. 16. Registration and password protection (restriction to content). 17. General disclaimers.      1. Advertisements distinctly labeled and separated from website content. 2. Disclosure of sponsorship. 3. Interaction or use of interactivity. |
| 4. CREDIBLE criteria [4] | Author | Yes |  | 1. Guidelines/standards of care. 2. Based on current research using MEDLINE searches. 3. Textbooks. 4. Disclosure of date of creation. 5. References. 6. General disclosures (educational, nonprofit, or commercial). 7. Disclosure of ownership. 8. Disclosure of sponsorship. 9. Disclosure of author affiliation. 10. Disclosure of authorship. 11. Inclusion of definition. 12. Epidemiology. 13. Etiology. 14. Diagnosis. 15. Management, with description of each treatment and how it works. 16. Complications. 17. Balance between benefits and harms. |
| 5. Criteria for the Selection of Health Information Sources Available on the Internet [5] | Latin American and Caribbean Center on Health Sciences Information | No | 1. Scope 2. Audience 3. Origin/ responsibility 4. Certification 5. Content 6. Access | 1. Inclusion of definition. 2. Target audience clearly defined. 3. Statement of purpose. 4. General disclosures (educational, nonprofit, or commercial). 5. Guidelines/standards of care. 6. Based on current research using MEDLINE searches. 7. Textbooks. 8. Expert consultation or personal opinion of the author. 9. Disclosure of date of last update. 10. Disclosure of date of creation. 11. Disclosure of sponsorship. 12. General disclaimers. 13. Data collection, who can access it, how it is accessed. 14. Layout with appropriate illustrations adjacent to the related text; visual cueing devices such as boxes, arrows, and shading used to direct attention to key content. 15. Does not require other computer applications for viewing or links are provided to download needed browser plug-in. 16. Registration and password protection (restriction to content). 17. Feedback mechanisms, response time for feedback, feature to rate the usefulness of information. 18. Easy navigation. 19. Relevant graphics and images. |
| 6. DARTS instrument [6] | Authors under Pharmaceut-ical Forum, Working Group on Information to Patients | Yes |  | 1. Disclosure of date of creation. 2. Disclosure of authorship. 3. Disclosure of author credentials. 4. References. 5. Sources clear. 6. General disclosures (educational, nonprofit, or commercial). 7. Disclosure of sponsorship. |
| 7. DISCERN [7] | Authors | Yes | 1. Reliable 2. Quality of information on treatment choices | 1. Statement of purpose. 2. Target audience clearly defined. 3. Management, with description of each treatment and how it works. 4. Self-management. 5. Balance between benefits and harms. 6. Complications. 7. Guidelines/standards of care. 8. Based on current research using MEDLINE searches. 9. Textbooks. 10. Expert consultation or personal opinion of the author. 11. Disclosure of date of last update. 12. References. 13. Disclosure of authorship. 14. Disclosure of sponsorship. 15. Coverage of areas of uncertainty. 16. Description of what would happen if no treatment is used. 17. Questions to discuss with those involved in the patient’s care. 18. Costs. |
| 8. Derived Reliability Scoring System [8] | Authors | No |  | 1. Disclosure of authorship. 2. Disclosure of author affiliation. 3. Disclosure of author credentials. 4. Disclosure of physician credentials. 5. References. 6. Disclosure of ownership. 7. Disclosure of sponsorship. 8. Disclosure of date of creation. 9. Statement of purpose. 10. Based on current research using MEDLINE searches. 11. Email address. 12. Advertisements distinctly labeled and separated from website content. |
| 9. eEUROPE 2002^a^ [9] | Commission of the European Communities | NR | 1. Transparency and honesty 2. Authority 3. Privacy and data protection 4. Updating of information 5. Accountability 6. Accessibility 7. Readability 8. Usability | 1. Disclosure of ownership. 2. Statement of purpose. 3. Target audience clearly defined. 4. Disclosure of sponsorship. 5. Disclosure of authorship. 6. Disclosure of author credentials. 7. Disclosure of physician credentials. 8. Disclosure of author affiliation. 9. Author is a recognized authority. 10. General disclaimers. 11. Disclosure of date of creation. 12. Feedback mechanisms, response time for feedback, feature to rate the usefulness of information. 13. Links provided with responsible partnering. 14. Editorial review process. 15. Available to people with disabilities or low-end technology. 16. Findability. 17. Internal search engine. |
| 10. eHealth Code of Ethics [10] | iHealthCoalition.org | No |  | 1. Disclosure of ownership. 2. Disclosure of sponsorship. 3. Disclosure of authorship. 4. General disclosures (educational, nonprofit, or commercial). 5. Disclosure of sponsorship. 6. Guidelines/standards of care. 7. Based on current research using MEDLINE searches. 8. Textbooks. 9. Expert consultation or personal opinion of the author. 10. Management, with description of each treatment and how it works. 11. Cultural match: appropriate language for the region. 12. Available to people with disabilities or low-end technology. 13. Disclosure of date of creation. 14. Sources clear. 15. References. 16. Data collection, who can access it, how it is accessed. 17. General disclaimers. 18. Statement of purpose. 19. Questions to discuss with those involved in the patient’s care. 20. Description of what would happen if no treatment is used. 21. Links provided with responsible partnering. 22. Message alert when leaving a secured website. 23. Feedback mechanisms, response time for feedback, feature to rate the usefulness of information. |
| 11. Evaluation and review of the ePrivacy directive [11] | European Commission | NR |  | 1. Message alert if cookies are used (with option to disable) 2. Data collection, who can access it, how it is accessed. 3. Option to opt in/out of subscription services. |
| 12. Evaluation criteria for medical websites^b^ [12] | Authors | Yes | 1. Evaluation of content 2. Organization of website | 1. General disclosures (educational, nonprofit, or commercial). 2. Target audience clearly defined. 3. Guidelines/standards of care. 4. Based on current research using MEDLINE searches. 5. Textbooks. 6. Expert consultation or personal opinion of the author. 7. Disclosure of authorship. 8. Disclosure of author credentials. 9. Disclosure of date of last update. 10. Disclosure of sponsorship. 11. Disclosure of author affiliation. 12. Author is a recognized authority. 13. Editorial review process. 14. Internal search engine. 15. Menu (directional icons, bars, indicators, listing, indexes). 16. References. 17. Links provided with responsible partnering. 18. Sources clear. 19. Visual aspect. 20. Option to download/print materials. 21. Large files include space for the size. 22. Email address. 23. Feedback mechanisms, response time for feedback, feature to rate the usefulness of information. 24. Cultural match: appropriate language for the region. |
| 13. Evaluation assessment for telehealth websites^c^ [13] | Authors | No | 1. Design assessment 2. Information assessment 3. Literacy assessment 4. Content assessment | 1. Browser compatibility. 2. Registration and password protection (restriction to content). 3. Layout with appropriate illustrations adjacent to the related text; visual cueing devices such as boxes, arrows, and shading used to direct attention to key content. 4. Relevant graphics and images. 5. Option to download/print materials. 6. Cultural match: appropriate language for the region. 7. Menu (directional icons, bars, indicators, listing, indexes). 8. Appropriate typography. 9. Internal search engine. 10. Entire page loads in less than 5 seconds. 11. Easy navigation. 12. Email address. 13. Fax number. 14. Feedback mechanisms, response time for feedback, feature to rate the usefulness of information. 15. Interaction or use of interactivity. 16. Type of material, cover images, illustrations, and media used to communicate. 17. Disclosure of authorship. 18. Statement of purpose. 19. Copyright. 20. General disclaimers. 21. References. 22. Disclosure of date of creation. 23. Use of a readability tool. 24. Layout with appropriate illustrations adjacent to the related text; visual cueing devices such as boxes, arrows, and shading used to direct attention to key content. 25. Subheadings and chunking. 26. Appropriate grammar. 27. Appropriate writing style. 28. Functionality (supports content, e.g., calculations). 29. Relevant graphics and images. 30. Cases/examples of desired behavior modeled or shown. |
| 14. Growth House [14] | Growthhouse.org | No |  | 1. Country. 2. Disclosure of sponsorship. 3. Advertisements distinctly labeled and separated from website content. 4. Interaction or use of interactivity. 5. Links provided with responsible partnering. 6. Easy navigation. 7. Entire page loads in less than 5 seconds. 8. Type of material, cover images, illustrations, and media used to communicate. |
| 15. Guidelines for AMA websites^b^ [15] | Authors | No | 1. Principles for content 2. Principles for   advertising and  sponsorship   1. Principles for privacy and confidentiality 2. Principles for   e-commerce | 1. Functionality (supports content, e.g., calculations). 2. Disclosure of ownership. 3. Disclosure of author affiliation. 4. Copyright. 5. Disclosure of sponsorship. 6. Browser compatibility. 7. Registration and password protection (restriction to content). 8. General disclaimers. 9. Editorial review process. 10. Disclosure of date of last update. 11. Disclosure of date of creation. 12. Appropriate writing style. 13. Cultural match: appropriate language for the region. 14. Appropriate grammar. 15. Sources clear. 16. Links provided with responsible partnering. 17. Easy navigation. 18. Does not require other computer applications for viewing or links are provided to download needed browser plug-in. 19. Option to download/print materials. 20. Feedback mechanisms, response time for feedback, feature to rate the usefulness of information. 21. Internal search engine. 22. Graphic files with “mouse over” indication of graphical content. 23. Large files include space for the size. 24. Email address. 25. Advertisements distinctly labeled and separated from website content. 26. Message alert when leaving a secured website. 27. Statement of purpose. 28. General disclosures (educational, nonprofit, or commercial). 29. General disclaimers. 30. Data collection, who can access it, how it is accessed. 31. Message alert if cookies are used (with option to disable). 32. Option to opt in/out of subscription services. 33. Email address. |
| 16. Guidelines for content creation and evaluation^b^ [16] | The Children's Partnership | No | 1. Authorship/   sponsorship/  purpose   1. Literacy level   of text   1. Accessibility to individuals with disability 2. Cultural focus of content 3. Cost of access of use 4. Geographic specificity of country 5. Source 6. Privacy 7. Informational quality 8. Presentation 9. Interactivity 10. Technical | 1. Disclosure of authorship. 2. General disclosures (educational, nonprofit, or commercial). 3. Disclosure of sponsorship. 4. Use of a readability tool. 5. Appropriate writing style. 6. Relevant graphics and images. 7. Appropriate sentence construction. 8. Cultural match: appropriate language for the region. 9. Type of material, cover images, illustrations, and media used to communicate. 10. Available to people with disabilities or low-end technology. 11. Appropriate color contrast. 12. Cultural match: images and examples presented in realistic and positive ways. 13. Country. 14. Disclosure of author credentials. 15. Disclosure of physician credentials. 16. Email address. 17. Fax number. 18. Feedback mechanisms, response time for feedback, feature to rate the usefulness of information. 19. Data collection, who can access it, how it is accessed. 20. Target audience clearly defined. 21. Appropriate grammar. 22. Copyright. 23. Disclosure of date of creation. 24. Sources clear. 25. Subheadings and chunking. 26. Layout with appropriate illustrations adjacent to the related text; visual cueing devices such as boxes, arrows, and shading used to direct attention to key content. 27. Easy navigation. 28. Appropriate typography. 29. Relevant graphics and images. 30. Visual aspect. 31. Does not require other computer applications for viewing or links are provided to download needed browser plug-in. 32. Appropriate color contrast. 33. Internal search engine. 34. Menu (directional icons, bars, indicators, listing, indexes). 35. Registration and password protection (restriction to content). 36. Interaction or use of interactivity. 37. Browser compatibility. 38. Option to download/print materials. 39. Disclosure of date of last update. |
| 17. Guidelines For Evaluating Web Sites [17] | Authors | No | 1. Authority 2. Currency 3. Objectivity/ accuracy 4. Structure 5. Readability | 1. Disclosure of authorship. 2. Disclosure of author credentials. 3. Email address. 4. General disclosures (educational, nonprofit, or commercial). 5. Disclosure of date of creation. 6. Links provided with responsible partnering. 7. Statement of purpose. 8. Author is a recognized authority. 9. Advertisements distinctly labeled and separated from website content. 10. References. 11. Sources clear. 12. Expert consultation or personal opinion of the author. 13. Easy navigation. 14. Internal search engine. 15. Use of a readability tool. 16. Subheadings and chunking. 17. Appropriate grammar. 18. Appropriate writing style. 19. Appropriate sentence construction. 20. Appropriate typography. 21. Relevant graphics and images. |
| 18. Health Literacy Online: a guide to writing and designing  easy-to-use health web sites^a^ [18] | US Department of Health and Human Services, Office of Disease  Prevention and Health Promotion | Yes |  | 1. Target audience clearly defined. 2. Cultural match: appropriate language for the region. 3. Easy navigation. 4. Subheadings and chunking. 5. Internal search engine. 6. Menu (directional icons, bars, indicators, listing, indexes). 7. Appropriate writing style. 8. Appropriate sentence construction. 9. Disclosure of date of creation. 10. Disclosure of authorship. 11. Disclosure of author credentials. 12. Email address. 13. Appropriate typography. 14. Visual aspect. 15. Relevant graphics and images. 16. Appropriate color contrast. 17. Available to people with disabilities or low-end technology. 18. Appropriate grammar. 19. Links provided with responsible partnering. 20. Browser compatibility. 21. Option to download/print materials. 22. Type of material, cover images, illustrations, and media used to communicate. 23. Interaction or use of interactivity. 24. Registration and password protection (restriction to content). 25. Layout with appropriate illustrations adjacent to the related text; visual cueing devices such as boxes, arrows, and shading used to direct attention to key content. |
| 19. Health website check-up service and accreditation program [19] | Utilization Review Accreditation Commission | No | 1. Disclosure 2. Health content and service delivery 3. Linking 4. Privacy and security 5. Accountability 6. Policies and procedures 7. Quality oversight committee 8. Health content and personal health management providers | 1. Disclosure of ownership. 2. General disclosures (educational, nonprofit, or commercial). 3. Email address. 4. Disclosure of sponsorship. 5. Links provided with responsible partnering. 6. Disclosure of physician credentials. 7. Disclosure of author credentials. 8. Disclosure of author affiliation. 9. Advertisements distinctly labeled and separated from website content. 10. Balance between benefits and harms. 11. Sources clear. 12. Disclosure of date of creation. 13. Self-management. 14. Questions to discuss with those involved in the patient’s care. 15. Message alert when leaving a secured website. 16. Registration and password protection (restriction to content). 17. Option to opt in/out of subscription services. 18. General disclaimers. 19. Feedback mechanisms, response time for feedback, feature to rate the usefulness of information. 20. Editorial review process. |
| 20. Health-Related Web Site Evaluation Form [20] | Authors | No | 1. Content 2. Authorship 3. Page aesthetics | 1. Guidelines/standards of care. 2. Based on current research using MEDLINE searches. 3. Textbooks. 4. Disclosure of date of creation. 5. References. 6. Sources clear. 7. Target audience clearly defined. 8. Use of a readability tool. 9. Disclosure of authorship. 10. Disclosure of ownership. 11. Disclosure of author credentials. 12. Disclosure of author affiliation. 13. Author is a recognized authority. 14. Layout with appropriate illustrations adjacent to the related text; visual cueing devices such as boxes, arrows, and shading used to direct attention to key content. 15. Menu (directional icons, bars, indicators, listing, indexes). 16. Internal search engine. 17. Links provided with responsible partnering. 18. Relevant graphics and images. 19. Advertisements distinctly labeled and separated from website content. 20. Type of material, cover images, illustrations, and media used to communicate. 21. Visual aspect. 22. Entire page loads in less than 5 seconds. 23. Interaction or use of interactivity. |
| 21. Healthcare Website Assessment Instrument [21] | Authors | Yes | 1. Content 2. Credibility 3. Navigability 4. Currency 5. Readability | 1. Disclosure of authorship. 2. Disclosure of ownership. 3. General disclosures (educational, nonprofit, or commercial). 4. Inclusion of definition. 5. Diagnosis. 6. Management, with description of each treatment and how it works. 7. Complications. 8. Monitoring. 9. Disclosure of author credentials. 10. Disclosure of physician credentials. 11. Sources clear. 12. References. 13. Guidelines/standards of care. 14. Based on current research using MEDLINE searches. 15. Textbooks. 16. Expert consultation or personal opinion of the author. 17. Links provided with responsible partnering. 18. Disclosure of date of last update. 19. Option to download/print materials. 20. Email address. 21. Feedback mechanisms, response time for feedback, feature to rate the usefulness of information. 22. Copyright. 23. Use of a readability tool. |
| 22. Heuristic Website Evaluation [22] | Authors | Yes | 1. Readability 2. Navigation 3. Content/   organization   1. Accessibility | 1. Appropriate sentence construction. 2. Appropriate typography. 3. Layout with appropriate illustrations adjacent to the related text; visual cueing devices such as boxes, arrows, and shading used to direct attention to key content. 4. Graphic files with “mouse over” indication of graphical content. 5. Menu (directional icons, bars, indicators, listing, indexes). 6. Easy navigation. 7. Internal search engine. 8. Findability. 9. Available to people with disabilities or low-end technology. 10. Links provided with responsible partnering. 11. Subheadings and chunking. 12. Appropriate writing style. 13. Appropriate color contrast. 14. Appropriate grammar. 15. Email address. 16. Feedback mechanisms, response time for feedback, feature to rate the usefulness of information. |
| 23. HON code [23] | Health On The Net Foundation | Yes | 1. Authoritative 2. Complementarity 3. Privacy 4. Attribution 5. Justifiability 6. Transparency 7. Financial disclosure 8. Advertising policy | 1. Disclosure of authorship. 2. Disclosure of author credentials. 3. Author is a recognized authority. 4. Editorial review process. 5. Statement of purpose. 6. General disclaimers. 7. Data collection, who can access it, how it is accessed. 8. Disclosure of date of creation. 9. Disclosure of date of last update. 10. Sources clear. 11. References. 12. Feedback mechanisms, response time for feedback, feature to rate the usefulness of information. 13. Email address. 14. Disclosure of sponsorship. 15. General disclosures (educational, nonprofit, or commercial). 16. Advertisements distinctly labeled and separated from website content. 17. Disclosure of ownership. |
| 24. Internet Medical Information Search [24] | Authors | No |  | 1. Cases/examples of desired behavior modeled or shown. 2. Management, with description of each treatment and how it works. 3. References. 4. Sources clear. 5. Based on current research using MEDLINE searches. 6. Textbooks. 7. Expert consultation or personal opinion of the author. |
| 25. Information quality instrument/HSWG/  Mitretek^a^ [25] | Health Summit Working Group | Yes | 1. Credibility 2. Content   (accuracy and completeness)   1. Disclosure 2. Links 3. Design   (accessibility)   1. Interactivity 2. Caveats | 1. Sources clear. 2. References. 3. Guidelines/standards of care. 4. Disclosure of date of last update. 5. Editorial review process. 6. Disclosure of sponsorship. 7. Disclosure of author affiliation. 8. Statement of purpose. 9. Data collection, who can access it, how it is accessed. 10. Links provided with responsible partnering. 11. Easy navigation. 12. Internal search engine. 13. Feedback mechanisms, response time for feedback, feature to rate the usefulness of information. 14. General disclosures (educational, nonprofit, or commercial). |
| 26. Interactivity code scheme [26] | Authors | No | 1. Accessibility 2. Navigation 3. Time 4. Personalized content 5. Delivery of message 6. Data entry and use 7. Entertainment 8. Promotions 9. Relationship | 1. Available to people with disabilities or low-end technology. 2. Does not require other computer applications for viewing or links are provided to download needed browser plug-in. 3. Menu (directional icons, bars, indicators, listing, indexes). 4. Links provided with responsible partnering. 5. Easy navigation. 6. Internal search engine. 7. Entire page loads in less than 5 seconds. 8. Relevant graphics and images. 9. Registration and password protection (restriction to content). 10. Appropriate typography. 11. Type of material, cover images, illustrations, and media used to communicate. 12. Feedback mechanisms, response time for feedback, feature to rate the usefulness of information. 13. Data collection, who can access it, how it is accessed. 14. Internal search engine. 15. Menu (directional icons, bars, indicators, listing, indexes). 16. Interaction or use of interactivity. 17. Advertisements distinctly labeled and separated from website content.      1. Email address. |
| 27. JAMA benchmark criteria [27] | Authors | Yes | 1. Authorship 2. Attribution 3. Disclosure 4. Currency | 1. Disclosure of authorship. 2. Disclosure of author affiliation. 3. Disclosure of author credentials. 4. Disclosure of physician credentials. 5. Author is a recognized authority. 6. References. 7. Sources clear. 8. Copyright. 9. Disclosure of ownership. 10. Disclosure of sponsorship. 11. Advertisements distinctly labeled and separated from website content. 12. General disclosures (educational, nonprofit, or commercial). 13. Links provided with responsible partnering. 14. Disclosure of date of creation. 15. Disclosure of date of last update. |
| 28. Judge Project^a^ [28] | Information Management  Research Institute, School of Informatics, North Umbria University | No |  | 1. General disclosures (educational, nonprofit, or commercial). 2. Statement of purpose. 3. Cultural match: appropriate language for the region. 4. Disclosure of sponsorship. 5. Disclosure of author affiliation. 6. Inclusion of definition. 7. Epidemiology. 8. Etiology. 9. Pathogenesis. 10. Clinical features. 11. Management, with description of each treatment and how it works. 12. Monitoring. 13. Complications. 14. Copyright. 15. Advertisements distinctly labeled and separated from website content. 16. Interaction or use of interactivity. 17. Layout with appropriate illustrations adjacent to the related text; visual cueing devices such as boxes, arrows, and shading used to direct attention to key content. 18. Available to people with disabilities or low-end technology. 19. Quality of visual presentation. 20. Type of material, cover images, illustrations, and media used to communicate. 21. Appropriate color contrast. 22. Disclosure of date of last update. 23. Internal search engine. 24. Links provided with responsible partnering. |
| 29. Lehigh University Web Resource Evaluation Checklist [29] | Lehigh University | No | 1. Authorship and authority 2. Accuracy 3. Objectivity and purpose 4. Currency of the website | 1. Disclosure of authorship. 2. Disclosure of author credentials. 3. Disclosure of sponsorship. 4. Author is a recognized authority. 5. Disclosure of author affiliation. 6. Email address. 7. Fax number. 8. Country. 9. Sources clear. 10. References. 11. Appropriate grammar. 12. Links provided with responsible partnering. 13. Relevant graphics and images. 14. Statement of purpose. 15. Balance between benefits and harms. 16. Appropriate writing style. 17. Appropriate sentence construction. 18. Advertisements distinctly labeled and separated from website content. 19. Disclosure of date of creation. 20. Disclosure of date of last update. |
| 30. LIDA [30] | Minervation.com | Yes | 1. Accessibility 2. Usability 3. Reliability | 1. Available to people with disabilities or low-end technology. 2. Layout with appropriate illustrations adjacent to the related text; visual cueing devices such as boxes, arrows, and shading used to direct attention to key content. 3. Registration and password protection (restriction to content). 4. Browser compatibility. 5. Dublin core tags. 6. Target audience clearly defined. 7. Appropriate grammar. 8. Appropriate typography. 9. Easy navigation. 10. Menu (directional icons, bars, indicators, listing, indexes). 11. Appropriate color contrast. 12. Subheadings and chunking. 13. Visual aspect. 14. Internal search engine. 15. Findability. 16. Does not require other computer applications for viewing or links are provided to download needed browser plug-in. 17. Graphic files with “mouse over” indication of graphical content. 18. Interaction or use of interactivity. 19. Feedback mechanisms, response time for feedback, feature to rate the usefulness of information. 20. Type of material, cover images, illustrations, and media used to communicate. 21. Disclosure of date of last update. 22. Disclosure of date of creation. 23. Disclosure of ownership. 24. Disclosure of sponsorship. 25. General disclosures (educational, nonprofit, or commercial). 26. Editorial review process. 27. Sources clear. 28. References. 29. Based on current research using MEDLINE searches. 30. Textbooks. 31. Expert consultation or personal opinion of the author. |
| 31. Making information accessible for all [31] | European Blind Union: the voice of blind and partially sighted people in Europe | NR |  | 1. Available to people with disabilities or low-end technology. 2. Subheadings and chunking. 3. Appropriate color contrast. 4. Type of material, cover images, illustrations, and media used to communicate. |
| 32. MedCERTAIN/  MedCIRCLE/  HIDDEL [32] | Collaboration for Internet Rating,  Certification, Labeling and Evaluation of Health  Information | NR |  | 1. Feedback mechanisms (accountability: user feedback), appropriate oversight responsibility (such as a named quality compliance officer for each site), response time for feedback, feature to rate the usefulness of information. 2. Email address. 3. Fax and phone number. 4. Disclosure of ownership. 5. Country. 6. Available to people with disabilities or low-end technology. 7. Disclosure of date of creation. 8. Disclosure of date of last update. 9. Disclosure of authorship. 10. Author is a recognized authority. 11. Cultural match: appropriate language for the region. 12. Statement of purpose with aim of website. 13. Dublin core tags. 14. Site statistics. 15. Target audience clearly defined. 16. Level of evidence. 17. Links provided with responsible partnering. 18. Disclosure of sponsorship. 19. Disclosure of author affiliation. 20. Disclosure of author credentials. 21. Sources clear. 22. Message alert when leaving a secured website. 23. Advertisements distinctly labeled and separated from website content. 24. General disclaimers. 25. Editorial review process. 26. References. 27. Clinical features. 28. Monitoring. 29. Complications. 30. Self-management. 31. Management, with description of each treatment and how it works. 32. Description of what would happen if no treatment is used. 33. Costs. 34. Findability. |
| 33. MedlinePlus Guide to Healthy Web Surfing and How to Write Easy-to-Read Health Materials [33] | NIH US National Library of Medicine | NR |  | 1. Target audience clearly defined. 2. Cultural match: appropriate language for the region. 3. Cultural match: images and examples presented in realistic and positive ways. 4. General disclosures (educational, nonprofit, or commercial). 5. Use of a readability tool. 6. Appropriate writing style. 7. Appropriate sentence construction. 8. Appropriate grammar. 9. Motivation. 10. Cases/examples of desired behavior modeled or shown. 11. Subheadings and chunking. 12. Editorial review process. 13. Balance between benefits and harms. 14. Appropriate typography. 15. Layout with appropriate illustrations adjacent to the related text; visual cueing devices such as boxes, arrows, and shading used to direct attention to key content. 16. Appropriate color contrast. 17. Type of material, cover images, illustrations, and media used to communicate. 18. Findability. 19. Menu (directional icons, bars, indicators, listing, indexes). 20. Quality of visual presentation. 21. Relevant graphics and images. 22. Available to people with disabilities or low-end technology. |
| 34. Netscoring: criteria to assess the quality of health internet information^a^ [34] | Association des Centraliens | No | 1. Credibility 2. Content   (accuracy)   1. Hyperlinks 2. Design 3. Interactivity 4. Quantitative   aspects   1. Ethics 2. Accessibility | 1. Disclosure of authorship. 2. Disclosure of author credentials. 3. Disclosure of sponsorship. 4. Disclosure of author affiliation. 5. Disclosure of physician credentials. 6. Author is a recognized authority. 7. Disclosure of date of creation. 8. Disclosure of date of last update. 9. Editorial review process. 10. General disclosures (educational, nonprofit, or commercial). 11. Cultural match: appropriate language for the region. 12. Hierarchy of evidence clear. 13. Sources clear. 14. References. 15. Menu (directional icons, bars, indicators, listing, indexes). 16. Entire page loads in less than 5 seconds. 17. Links provided with responsible partnering. 18. Use of a readability tool. 19. Option to download/print materials. 20. Layout with appropriate illustrations adjacent to the related text; visual cueing devices such as boxes, arrows, and shading used to direct attention to key content. 21. Visual aspect. 22. Feedback mechanisms, response time for feedback, feature to rate the usefulness of information. 23. Email address. 24. Message alert if cookies are used (with option to disable). 25. Interaction or use of interactivity. 26. Site statistics. 27. Internal search engine. |
| 35. Nursing website evaluation questionnaire^a^ [35] | Authors | Yes | 1. Overall impression 2. Download and   switch speed   1. Accessibility and   convenience   1. Web page content   (completeness)   1. Services provided   by website   1. Browser compatibility | 1. Statement of purpose. 2. Layout with appropriate illustrations adjacent to the related text; visual cueing devices such as boxes, arrows, and shading used to direct attention to key content. 3. Quality of visual presentation. 4. Entire page loads in less than 5 seconds. 5. Internal search engine. 6. Relevant graphics and images. 7. Links provided with responsible partnering. 8. Available to people with disabilities or low-end technology. 9. Menu (directional icons, bars, indicators, listing, indexes). 10. Disclosure of date of creation. 11. Disclosure of date of last update. 12. Sources clear. 13. Interaction or use of interactivity. 14. Easy navigation. 15. Disclosure of author credentials. 16. Author is a recognized authority. 17. Email address. 18. Disclosure of authorship. 19. Browser compatibility. |
| 36. Organizing medical networked information  (OMNI)^a b d^ [36] | OMNI Advisory Group | No | 1. Audience 2. Authority 3. Provenance 4. Content evaluation   (accuracy)   1. Coverage   (Completeness)   1. Accuracy of information content 2. Uniqueness/ comparison with other sources 3. Accessibility and usability | 1. Target audience clearly defined. 2. Disclosure of authorship. 3. Disclosure of author credentials. 4. Author is a recognized authority. 5. Copyright. 6. Disclosure of sponsorship. 7. Disclosure of ownership. 8. Email address. 9. References. 10. Sources clear. 11. Disclosure of physician credentials. 12. Disclosure of date of creation. 13. Option to download/print materials. 14. Date of technical maintenance. 15. Disclosure of date of last update. 16. Links provided with responsible partnering. 17. Guidelines/standards of care. 18. Based on current research using MEDLINE searches. 19. Textbooks. 20. Expert consultation or personal opinion of the author. 21. Browser compatibility. 22. Interaction or use of interactivity. 23. Available to people with disabilities or low-end technology. 24. Large files include space for the size. 25. Relevant graphics and images. 26. Date of technical maintenance. 27. Country. 28. Registration and password protection (restriction to content). 29. Cultural match: appropriate language for the region. 30. Layout with appropriate illustrations adjacent to the related text; visual cueing devices such as boxes, arrows, and shading used to direct attention to key content. 31. Menu (directional icons, bars, indicators, listing, indexes). 32. Internal search engine. 33. Visual aspect. 34. Quality of visual presentation. 35. Entire page loads in less than 5 seconds. 36. Type of material, cover images, illustrations, and media used to communicate. 37. Easy navigation. 38. Interaction or use of interactivity. 39. Feedback mechanisms, response time for feedback, feature to rate the usefulness of information. 40. Fax number. |
| 37. Oncology Nursing Society [37] | Oncology Nursing Society | No |  | 1. Disclosure of authorship. 2. Disclosure of author credentials. 3. Author is a recognized authority. 4. Disclosure of sponsorship. 5. Disclosure of date of creation. 6. General disclaimers. |
| 38. Overall quality score [38] | Authors | No | 1. Accuracy 2. Reliability 3. Depth | 1. Guidelines/standards of care. 2. Disclosure of sponsorship. 3. Disclosure of author affiliation. 4. Sources clear. 5. Target audience clearly defined. 6. Disclosure of authorship. 7. References. 8. Sources clear. |
| 39. PEMAT instrument [39] | Authors for Agency for Healthcare Research and Quality | Yes | 1. Understandability 2. Actionability | 1. General disclosures (educational, nonprofit, or commercial). 2. Appropriate grammar. 3. Appropriate sentence construction. 4. Subheadings and chunking. 5. Menu (directional icons, bars, indicators, listing, indexes). 6. Layout with appropriate illustrations adjacent to the related text visual cueing devices such as boxes, arrows, and shading used to direct attention to key content. 7. Appropriate typography. 8. Type of material, cover images, illustrations, and media used to communicate. 9. Statement of purpose. 10. Subheadings and chunking. 11. Relevant graphics and images. 12. Motivation. |
| 40. PLEASED criteria^a^ [40] | Author | No | 1. Purpose 2. Links 3. Editorial (site content; accuracy) 4. Author 5. Site 6. Ethical 7. Date | 1. General disclosures (educational, nonprofit, or commercial). 2. Links provided with responsible partnering. 3. Target audience clearly defined. 4. Appropriate grammar. 5. Disclosure of sponsorship. 6. Disclosure of author affiliation. 7. Disclosure of date of creation. 8. Disclosure of authorship. 9. Disclosure of author credentials. 10. Email address. 11. Easy navigation. 12. Layout with appropriate illustrations adjacent to the related text; visual cueing devices such as boxes, arrows, and shading used to direct attention to key content. 13. Entire page loads in less than 5 seconds. 14. Relevant graphics and images. 15. Disclosure of ownership. 16. Findability. 17. Disclosure of date of last update. 18. Disclosure of date of creation. |
| 41. Protecting electronic health information [41] | Committee on Maintaining Privacy and Security in  Healthcare Applications of the National Information Infrastructure | NR |  | 1. Data collection, who can access it, how it is accessed. 2. General disclaimers. |
| 42. Publication quality criteria^a^ [42] | Authors | No | 1. Content   (accuracy)   1. Authorship 2. Source 3. Attribution 4. Readership 5. Ease of use (usability) 6. Design 7. Age of study 8. Access   10 -Disclosure | 1. Disclosure of authorship. 2. Disclosure of author affiliation. 3. Disclosure of author credentials. 4. References. 5. Sources clear. 6. Target audience clearly defined. 7. Easy navigation. 8. Functionality (supports content, e.g., calculations). 9. Layout with appropriate illustrations adjacent to the related text visual cueing devices such as boxes, arrows, and shading used to direct attention to key content. 10. Appropriate color contrast. 11. Visual aspect. 12. Disclosure of date of creation. 13. Registration and password protection (restriction to content). 14. Disclosure of ownership. 15. Disclosure of sponsorship. |
| 43. Quality Checklist [43] | Authors | Yes | 1. Authorship 2. Content 3. Currency 4. Usefulness 5. Disclosure 6. User support 7. Privacy and confidentiality | 1. Disclosure of authorship 2. Disclosure of author credentials. 3. Sources clear. 4. References. 5. Disclosure of date of creation. 6. Target audience clearly defined.      1. Disclosure of ownership. 2. Disclosure of sponsorship. 3. Feedback mechanisms, response time for feedback, feature to rate the usefulness of information. 4. Email address. 5. Registration and password protection (restriction to content). 6. General disclaimers. |
| 44. Quality component scoring system, Technical component scoring system [44] | Authors | No | 1. Authorship 2. Attribution 3. Sponsorship 4. Currency | 1. Disclosure of authorship. 2. Disclosure of author credentials. 3. Fax number. 4. Email address. 5. Copyright. 6. References. 7. Sources clear. 8. Disclosure of sponsorship. 9. Disclosure of ownership. 10. General disclosures (educational, nonprofit, or commercial). 11. Disclosure of date of creation. 12. Disclosure of date of last update. |
| 45. Quality Evaluation Instrument, Integrity Score [45] | Authors | Yes |  | 1. Inclusion of definition. 2. Epidemiology. 3. Etiology. 4. Pathogenesis. 5. Clinical features. 6. Diagnosis. 7. Management, with description of each treatment and how it works. 8. Complications. 9. Balance between benefits and harms. 10. Costs. 11. Motivation. 12. Use of a readability tool. 13. General disclosures (educational, nonprofit, or commercial). 14. Disclosure of authorship. 15. Disclosure of date of creation. 16. Disclosure of date of last update. 17. Disclosure of sponsorship. 18. Links provided with responsible partnering. 19. General disclaimers. 20. Data collection, who can access it, how it is accessed. |
| 46. Quality score/Hogne-Sandvik [46] | Author | No |  | 1. Disclosure of ownership. 2. General disclosures (educational, nonprofit, or commercial). 3. Disclosure of authorship. 4. Disclosure of author credentials. 5. Sources clear. 6. References. 7. Interaction or use of interactivity. 8. Email address. 9. Disclosure of date of creation. 10. Easy navigation. 11. Internal search engine. 12. Disclosure of sponsorship. 13. Disclosure of author affiliation. 14. Interaction or use of interactivity. |
| 47. Quality standards for medical publishing on the web [47] | British Healthcare Internet Association | No |  | 1. Target audience clearly defined. 2. References. 3. Option to download/print materials. 4. Statement of purpose. 5. Disclosure of date of creation. 6. Feedback mechanisms, response time for feedback, feature to rate the usefulness of information. 7. Email address. 8. Disclosure of author affiliation. 9. Disclosure of sponsorship. 10. Copyright. |
| 48. Questionnaire to evaluate  health websites according  to European criteria [48] | Authors | Yes | 1. Transparency and absence of conflict of interest 2. Authorship 3. Personal data protection 4. Responsibility 5. Accessibility | 1. Disclosure of authorship. 2. Author is a recognized authority. 3. Disclosure of ownership. 4. Email address. 5. Country. 6. Statement of purpose. 7. Target audience clearly defined. 8. Disclosure of sponsorship. 9. Sources clear. 10. Disclosure of date of creation. 11. General disclaimers. 12. Data collection, who can access it, how it is accessed. 13. Feedback mechanisms, response time for feedback, feature to rate the usefulness of information. 14. Editorial review process. 15. Links provided with responsible partnering. 16. Findability. 17. Internal search engine. 18. Appropriate color contrast. 19. Appropriate typography. 20. Cultural match: appropriate language for the region. 21. Available to people with disabilities or low-end technology. |
| 49. Quick information evaluation instrument [49] | Author | No |  | 1. Disclosure of ownership. 2. Sources clear. 3. References. 4. Sources clear. 5. Based on current research using MEDLINE searches. 6. Guidelines/standards of care. 7. Disclosure of date of creation. 8. Disclosure of author credentials. 9. Disclosure of physician credentials. 10. General disclosures (educational, nonprofit, or commercial). 11. Email address. 12. Fax number. |
| 50. QUICK [50] | Quick.org | No |  | 1. Disclosure of authorship. 2. Author is a recognized authority. 3. Email address. 4. General disclosures (educational, nonprofit, or commercial). 5. Target audience clearly defined. 6. Findability. 7. Disclosure of author credentials. 8. Sources clear. 9. References. 10. Disclosure of date of creation. 11. Disclosure of date of last update. 12. Disclosure of sponsorship. 13. Disclosure of author affiliation. 14. Balance between benefits and harms. |
| 51. SAM instrument [51] | Authors | Yes | 1. Content 2. Literacy demand 3. Graphics 4. Layout and typography 5. Learning stimulation and motivation 6. Cultural appropriateness | 1. General disclosures (educational, nonprofit, or commercial). 2. Cases/examples of desired behavior modeled or shown. 3. Statement of purpose. 4. Use of a readability tool. 5. Appropriate writing style. 6. Appropriate grammar. 7. Appropriate sentence construction. 8. Road signs. 9. Type of material, cover images, illustrations, and media used to communicate. 10. Relevant graphics and images. 11. Graphic files with “mouse over” indication of graphical content. 12. Layout with appropriate illustrations adjacent to the related text; visual cueing devices such as boxes, arrows, and shading used to direct attention to key content. 13. Appropriate color contrast. 14. Appropriate typography. 15. Subheadings and chunking. 16. Interaction or use of interactivity. 17. Motivation. 18. Cultural match: appropriate language for the region. 19. Cultural match: images and examples presented in realistic and positive ways. |
| 52. Self-assessment method for evaluation of websites (Jones Instrument) [52] | Author | Yes | 1. Content   (accuracy)   1. Design 2. Communication 3. Credibility | 1. Quality of visual presentation. 2. Appropriate writing style. 3. Layout with appropriate illustrations adjacent to the related text; visual cueing devices such as boxes, arrows, and shading used to direct attention to key content. 4. Type of material, cover images, illustrations, and media used to communicate. 5. Relevant graphics and images. 6. Appropriate writing style. 7. Appropriate sentence construction. 8. Findability. 9. Easy navigation. 10. Disclosure of authorship. 11. Author is a recognized authority. 12. Disclosure of author credentials. 13. Disclosure of date of creation. 14. Feedback mechanisms, response time for feedback, feature to rate the usefulness of information. |
| 53. SPAT website evaluation instrument [53] | Spat.pitt.edu | No |  | 1. General disclosures (educational, nonprofit, or commercial). 2. Sources clear. 3. Disclosure of ownership. 4. Target audience clearly defined. 5. Disclosure of date of creation. |
| 54. Stanford Guidelines for Web Credibility [54] | Stanford Web Credibility Research | No |  | 1. References. 2. Sources clear. 3. Links provided with responsible partnering. 4. Email address. 5. Disclosure of authorship. 6. Disclosure of author credentials. 7. Author is a recognized authority. 8. Expert consultation or personal opinion of the author. 9. Fax number. 10. Layout with appropriate illustrations adjacent to the related text; visual cueing devices such as boxes, arrows, and shading used to direct attention to key content. 11. Appropriate typography. 12. Visual aspect. 13. Quality of visual presentation. 14. Disclosure of date of creation. 15. Disclosure of date of last update. 16. Advertisements distinctly labeled and separated from website content. 17. Country. 18. Disclosure of authorship. 19. Disclosure of sponsorship. 20. Disclosure of author credentials. 21. Author is a recognized authority. 22. Disclosure of ownership. |
| 55. The 5 Cs website evaluation instrument^a^ [55] | Author | No | 1. Credibility 2. Currency 3. Content 4. Construction 5. Clarity | 1. Disclosure of authorship. 2. Disclosure of author credentials. 3. Author is a recognized authority. 4. General disclosures (educational, nonprofit, or commercial). 5. References. 6. Disclosure of date of creation. 7. Disclosure of date of last update. 8. Links provided with responsible partnering. 9. Sources clear. 10. Appropriate writing style. 11. Textbooks. 12. Guidelines/standards of care. 13. Expert consultation or personal opinion of the author. 14. Based on current research using MEDLINE searches. 15. Visual aspect. 16. Quality of visual presentation. 17. Layout with appropriate illustrations adjacent to the related text visual cueing devices such as boxes, arrows, and shading used to direct attention to key content. 18. Menu (directional icons, bars, indicators, listing, indexes). 19. Findability. 20. Internal search engine. 21. Subheadings and chunking. 22. Appropriate color contrast. 23. Appropriate typography. 24. Advertisements distinctly labeled and separated from website content. 25. Relevant graphics and images. 26. Registration and password protection (restriction to content). 27. Does not require other computer applications for viewing or links are provided to download needed browser plug-in. 28. Appropriate grammar. 29. Target audience clearly defined. 30. Cultural match: appropriate language for the region. 31. Available to people with disabilities or low-end technology. |
| 56. The UsabAIPO Heuristic Method [56] | Authors | No | 1. Usability | 1. Appropriate color contrast. 2. Appropriate typography. 3. Relevant graphics and images. 4. Easy navigation. 5. Graphic files with “mouse over” indication of graphical content. 6. Interaction or use of interactivity. 7. Does not require other computer applications for viewing or links are provided to download needed browser plug-in. 8. Links provided with responsible partnering. 9. Menu (directional icons, bars, indicators, listing, indexes). 10. Statement of purpose. 11. Disclosure of date of creation. 12. Cultural match: appropriate language for the region. 13. Internal search engine. 14. Findability. 15. Visual aspect. 16. Type of material, cover images, illustrations, and media used to communicate. |
| 57. Usability engineering [57] | Author | Yes |  | 1. Relevant graphics and images. 2. Appropriate color contrast. 3. Cultural match: appropriate language for the region. 4. Menu (directional icons, bars, indicators, listing, indexes). 5. Layout with appropriate illustrations adjacent to the related text; visual cueing devices such as boxes, arrows, and shading used to direct attention to key content. 6. Feedback mechanisms, response time for feedback, feature to rate the usefulness of information. 7. Internal search engine. 8. Option to opt in/out of subscription services. |
| 58. Web and Usability Guidelines [58] | US Department of Health & Human Services | Yes | 1. Optimizing the user experience 2. Accessibility 3. Hardware and software 4. The homepage 5. Page layout 6. Navigation 7. Scrolling and paging 8. Headings, titles, and labels 9. Text appearance 10. Graphics, images, and multimedia 11. Writing web content 12. Content organization 13. Search | 1. Relevant graphics and images. 2. Entire page loads in less than 5 seconds. 3. Layout with appropriate illustrations adjacent to the related text; visual cueing devices such as boxes, arrows, and shading used to direct attention to key content. 4. Option to download/print materials. 5. Appropriate grammar. 6. Large files include space for the size. 7. Feedback mechanisms, response time for feedback, feature to rate the usefulness of information. 8. Email address. 9. Site statistics. 10. Appropriate color contrast. 11. Easy navigation. 12. Does not require other computer applications for viewing or links are provided to download needed browser plug-in. 13. Available to people with disabilities or low-end technology. 14. Type of material, cover images, illustrations, and media used to communicate. 15. Browser compatibility. 16. Quality of visual presentation. 17. Visual aspect. 18. General disclosures (educational, nonprofit, or commercial). 19. Statement of purpose. 20. Date of technical maintenance. 21. Road signs.      1. Subheadings and chunking. 2. Hierarchy of evidence clear. 3. Appropriate typography. 4. Graphic files with “mouse over” indication of graphical content. 5. Country. 6. Menu (directional icons, bars, indicators, listing, indexes). 7. Links provided with responsible partnering. 8. Appropriate writing style. 9. Appropriate sentence construction. 10. Road signs. 11. Type of material, cover images, illustrations, and media used to communicate. 12. Relevant graphics and images. 13. Internal search engine. |
| 59. Webmedqual Scale^b^ [59] | Authors | Yes | 1. Content 2. Authority of source 3. Design 4. Accessibility and availability 5. User support 6. Confidentiality 7. E-commerce-optional criteria | 1. Expert consultation or personal opinion of the author. 2. Questions to discuss with those involved in the patient’s care. 3. Balance between benefits and harms. 4. Editorial review process. 5. General disclosures (educational, nonprofit, or commercial). 6. Disclosure of date of creation. 7. Disclosure of date of last update. 8. References. 9. Based on current research using MEDLINE searches. 10. Textbooks. 11. Guidelines/standards of care. 12. Target audience clearly defined. 13. Disclosure of authorship. 14. Disclosure of ownership. 15. Author is a recognized authority. 16. Disclosure of sponsorship. 17. Disclosure of author affiliation. 18. Copyright. 19. Interaction or use of interactivity. 20. Easy navigation. 21. Layout with appropriate illustrations adjacent to the related text; visual cueing devices such as boxes, arrows, and shading used to direct attention to key content. 22. Visual aspect. 23. Quality of visual presentation. 24. Findability. 25. Relevant graphics and images. 26. Type of material, cover images, illustrations, and media used to communicate. 27. Does not require other computer applications for viewing or links are provided to download needed browser plug-in. 28. Option to download/print materials. 29. Large files include space for the size.      1. Cultural match: appropriate language for the region. 2. Registration and password protection (restriction to content). 3. Available to people with disabilities or low-end technology. 4. Links provided with responsible partnering. 5. Feedback mechanisms, response time for feedback, feature to rate the usefulness of information. 6. Email address. 7. Fax number. 8. Country. 9. References. 10. Data collection, who can access it, how it is accessed. 11. Option to opt in/out of subscription services. 12. Message alert when leaving a secured website. 13. General disclaimers. 14. Message alert if cookies are used (with option to disable). |
| 60. Web Evaluation Criteria [60] | Authors | No | 1. Website construction 2. Website   operation   1. Website   accessibility   1. Website   content | 1. Data collection, who can access it, how it is accessed. 2. Disclosure of authorship. 3. Disclosure of author credentials. 4. Email address. 5. General disclosures (educational, nonprofit, or commercial). 6. Feedback mechanisms, response time for feedback, feature to rate the usefulness of information. 7. Disclosure of date of creation. 8. Disclosure of date of last update. 9. Links provided with responsible partnering. 10. Easy navigation. 11. Internal search engine. 12. General disclosures (educational, nonprofit, or commercial). 13. Guidelines/standards of care. 14. Based on current research using MEDLINE searches. 15. Textbooks. 16. Expert consultation or personal opinion of the author. |
| 61. Web consortium guidelines [61] | World Wide Web Consortium | NR |  | 1. Available to people with disabilities or low-end technology. 2. Easy navigation. 3. Findability. 4. Appropriate color contrast. 5. Appropriate typography. 6. Cultural match: appropriate language for the region. 7. Use of a readability tool. 8. Appropriate grammar. 9. Appropriate writing style. 10. Appropriate sentence construction. 11. Type of material, cover images, illustrations, and media used to communicate. 12. Feedback mechanisms, response time for feedback, feature to rate the usefulness of information. 13. Email address. 14. Menu (directional icons, bars, indicators, listing, indexes). 15. Links provided with responsible partnering. |
| 62. Website and quality Indicators [62] | Authors | No |  | 1. Disclosure of authorship. 2. Disclosure of ownership. 3. Disclosure of sponsorship. 4. Disclosure of author affiliation. 5. Disclosure of date of creation. 6. Author is a recognized authority. 7. Guidelines/standards of care. 8. Based on current research using MEDLINE searches. 9. Feedback mechanisms, response time for feedback, feature to rate the usefulness of information. 10. Email address. 11. Fax number. 12. Links provided with responsible partnering. 13. Easy navigation. 14. Appropriate typography. 15. Option to download/print materials. 16. Internal search engine. 17. Editorial review process. 18. Site statistics. |
| 63. WebWatch^b^ [63] | Consumer union policy and action from consumer reports | No | 1. Identity 2. Advertising and sponsorships 3. Customer Service 4. Corrections 5. Privacy | 1. Country. 2. Fax number. 3. Feedback mechanisms, response time for feedback, feature to rate the usefulness of information. 4. Disclosure of ownership. 5. General disclosures (educational, nonprofit, or commercial). 6. Advertisements distinctly labeled and separated from website content. 7. Disclosure of sponsorship. 8. Disclosure of author affiliation. 9. Disclosure of date of creation. 10. General disclaimers. 11. Data collection, who can access it, how it is accessed. 12. Message alert if cookies are used (with option to disable). 13. Data collection, who can access it, how it is accessed. |
| 64. Darmoni Criteria^d^ [64] |  | No |  | 1. Author is a recognized authority. 2. Disclosure of date of creation. 3. Disclosure of authorship. 4. General disclosures (educational, nonprofit, or commercial). 5. Statement of purpose. 6. Editorial review process. 7. Disclosure of ownership. 8. Feedback mechanisms, response time for feedback, feature to rate the usefulness of information. 9. Guidelines/standards of care. 10. Based on current research using MEDLINE searches. 11. Textbooks. 12. Expert consultation or personal opinion of the author. 13. Internal search engine. 14. Menu (directional icons, bars, indicators, listing, indexes). 15. Easy navigation. 16. References. 17. Links provided with responsible partnering. 18. Site statistics. |
| 65. Health Promotion Instrument^a^ ^d^ [43] |  | No | 1. Authorship 2. Content 3. Accuracy 4. Authority 5. Currency 6. Audience 7. Accessibility 8. User support   and feedback   1. External links | 1. Disclosure of authorship. 2. Expert consultation or personal opinion of the author. 3. General disclosures (educational, nonprofit, or commercial). 4. Balance between benefits and harms. 5. Statement of purpose. 6. Disclosure of ownership. 7. Disclosure of author credentials. 8. Disclosure of date of creation. 9. Target audience clearly defined. 10. Easy navigation. 11. Available to people with disabilities or low-end technology. 12. Internal search engine. 13. Email address. 14. Feedback mechanisms, response time for feedback, feature to rate the usefulness of information. 15. Country. 16. Links provided with responsible partnering. |
| 66. Ansani et al [65] | Authors | Yes |  | 1. Disclosure of ownership. 2. Disclosure of sponsorship. 3. Disclosure of author affiliation. 4. Statement of purpose. 5. Advertisements distinctly labeled and separated from website content. 6. References. 7. Disclosure of author credentials. 8. Feedback mechanisms, response time for feedback, feature to rate the usefulness of information. 9. Email address. 10. Disclosure of date of creation. 11. Editorial review process. 12. Easy navigation. 13. Internal search engine. 14. Available to people with disabilities or low-end technology. |
| 67. Aslani et al [66] | Authors | No |  | 1. Disclosure of authorship. 2. Disclosure of ownership. 3. Editorial review process. 4. Author is a recognized authority. 5. Disclosure of sponsorship. 6. Advertisements distinctly labeled and separated from website content. 7. General disclosures (educational, nonprofit, or commercial). 8. Statement of purpose. 9. Sources clear. 10. References. 11. Use of a readability tool. 12. Appropriate writing style. 13. Appropriate sentence construction. 14. Type of material, cover images, illustrations, and media used to communicate. 15. Findability. 16. Internal search engine. 17. Links provided with responsible partnering. 18. General disclaimers. 19. Email address. 20. Feedback mechanisms, response time for feedback, feature to rate the usefulness of information. 21. Disclosure of date of last update. |
| 68. Fricke [67] | Authors | No | 1. Accuracy | 1. Clinical features. 2. Pathogenesis. 3. Management, with description of each treatment and how it works. |
| 69. Smart [68] | Authors | No |  | 1. Links provided with responsible partnering. 2. Visual aspect. 3. Email address. 4. Balance between benefits and harms. 5. Monitoring. 6. Use of a readability tool. |
| 70. Smith [69] | Author | No | 1. Content 2. Structure 3. Presentations 4. Graphics 5. Speed 6. Interactivity 7. Dynamism, freebies,   registration   1. Servers | 1. Disclosure of date of creation. 2. Links provided with responsible partnering. 3. Menu (directional icons, bars, indicators, listing, indexes). 4. Easy navigation. 5. Option to download/print materials. 6. Appropriate color contrast. 7. Layout with appropriate illustrations adjacent to the related text; visual cueing devices such as boxes, arrows, and shading used to direct attention to key content. 8. Relevant graphics and images. 9. Entire page loads in less than 5 seconds. 10. Interaction or use of interactivity. 11. Registration and password protection (restriction to content). 12. Dublin core tags.      1. Disclosure of date of last update. 2. General disclaimers. |
| 71. Seomun et al [70] | Authors | No | 1. Website construction 2. Website   operation   1. Website   accessibility   1. Website content | 1. General disclaimers. 2. Disclosure of authorship. 3. Author is a recognized authority. 4. Email address. 5. Easy navigation. 6. Disclosure of date of creation. 7. Disclosure of date of last update. 8. Links provided with responsible partnering. 9. Internal search engine. 10. General disclosures (educational, nonprofit, or commercial). 11. Statement of purpose. 12. Layout with appropriate illustrations adjacent to the related text; visual cueing devices such as boxes, arrows, and shading used to direct attention to key content. |
| 72. Schloman et al^a^ [71] | Authors | No | 1. Creator of website 2. Purpose and intention of website 3. Accuracy of information 4. Currency of information 5. Design of website (readability) | 1. Author is a recognized authority. 2. Disclosure of author credentials. 3. Disclosure of author affiliation. 4. Email address. 5. General disclosures (educational, nonprofit, or commercial). 6. Easy navigation. 7. Statement of purpose. 8. Target audience clearly defined. 9. Hierarchy of evidence clear. 10. Disclosure of sponsorship. 11. Editorial review process. 12. References. 13. Sources clear. 14. Links provided with responsible partnering. 15. Disclosure of date of creation. 16. Disclosure of date of last update. 17. Cultural match: appropriate language for the region. 18. Entire page loads in less than 5 seconds. 19. Does not require other computer applications for viewing or links are provided to download needed browser plug-in. 20. Appropriate grammar. |
| 73. Wyatt^a^ [72] | Author | No | 1. Credibility, conflicts of interest 2. Structure and content of web site 3. Functions of web site 4. Impact of web site | 1. Disclosure of ownership. 2. Disclosure of sponsorship. 3. Disclosure of authorship. 4. Disclosure of author credentials. 5. References. 6. Sources clear. 7. Use of a readability tool. 8. Links provided with responsible partnering. 9. Type of material, cover images, illustrations, and media used to communicate. 10. Internal search engine. 11. Easy navigation. 12. Statement of purpose. 13. Feedback mechanisms, response time for feedback, feature to rate the usefulness of information. |
| 74. Whitten et al [73] | Authors | No |  | 1. Relevant graphics and images. 2. Internal search engine. 3. Option to download/print materials. 4. Appropriate color contrast. 5. Appropriate typography. 6. Appropriate writing style. 7. Easy navigation. 8. Links provided with responsible partnering. 9. Advertisements distinctly labeled and separated from website content. 10. Copyright. 11. General disclaimers. 12. References. 13. Disclosure of authorship. 14. Disclosure of sponsorship. 15. Disclosure of date of creation. 16. Motivation. 17. Inclusion of definition. 18. Diagnosis. 19. Clinical features. 20. Management, with description of each treatment and how it works. 21. Cases/examples of desired behavior modeled or shown. 22. Self-management. 23. Use of a readability tool. 24. Appropriate sentence construction. 25. Appropriate grammar. 26. Cultural match: appropriate language for the region. |
| 75. Alzheimer disease information instrument [74] | Authors | No | 1. General details 2. Information for carers 3. Currency 4. Using the web site (usability) | 1. Disclosure of authorship. 2. Disclosure of ownership. 3. Option to download/print materials. 4. Clinical features. 5. Management, with description of each treatment and how it works. 6. Monitoring. 7. Complications. 8. Self-management. 9. Questions to discuss with those involved in the patient’s care. 10. Balance between benefits and harms. 11. Interaction or use of interactivity. 12. Disclosure of date of creation. 13. Disclosure of date of last update. 14. Findability. 15. Links provided with responsible partnering. 16. Internal search engine. 17. Email address. 18. Feedback mechanisms, response time for feedback, feature to rate the usefulness of information. 19. Available to people with disabilities or low-end technology. 20. General disclaimers. |
| 76. Bipolar Website Quality Checklist [75] | Authors | No | 1. Credibility 2. Currency 3. Objectivity 4. Availability and usability 5. Design and aesthetics 6. Breadth and accuracy | 1. Disclosure of author credentials. 2. Feedback mechanisms, response time for feedback, feature to rate the usefulness of information. 3. Email address. 4. Sources clear. 5. Disclosure of date of creation. 6. General disclosures (educational, nonprofit, or commercial). 7. Target audience clearly defined. 8. Disclosure of sponsorship. 9. Disclosure of author affiliation. 10. Advertisements distinctly labeled and separated from website content. 11. Balance between benefits and harms. 12. Coverage of areas of uncertainty. 13. Links provided with responsible partnering. 14. Internal search engine. 15. Easy navigation. 16. Relevant graphics and images. 17. Visual aspect. 18. Appropriate typography. 19. Clinical features. 20. Etiology. 21. Complications. 22. Management, with description of each treatment and how it works. 23. Self-management. 24. Description of what would happen if no treatment is used. 25. Motivation. 26. Cases/examples of desired behavior modeled or shown. 27. Guidelines/standards of care. 28. Based on current research using MEDLINE searches. 29. Textbooks. 30. Expert consultation or personal opinion of the author. |
| 77. Criteria for evaluation of neuropathology websites [76] | Authors | No | 1. General 2. Scientific merits 3. Technical merits | 1. Target audience clearly defined. 2. Guidelines/standards of care. 3. Based on current research using MEDLINE searches. 4. Textbooks. 5. Expert consultation or personal opinion of the author. 6. Interaction or use of interactivity. 7. Easy navigation. 8. Sources clear. 9. Disclosure of authorship. 10. Disclosure of ownership. 11. Disclosure of sponsorship. 12. Relevant graphics and images. 13. Layout with appropriate illustrations adjacent to the related text; visual cueing devices such as boxes, arrows, and shading used to direct attention to key content. 14. Appropriate writing style. 15. Balance between benefits and harms. 16. Links provided with responsible partnering. 17. Disclosure of date of creation. 18. Sources clear. 19. References. 20. Visual aspect. 21. Quality of visual presentation. 22. Internal search engine. 23. Email address. 24. Feedback mechanisms, response time for feedback, feature to rate the usefulness of information. |
| 78. Female urinary incontinence [77] | Authors | No | 1. Developer 2. Author and sources 3. Interactivity and navigability 4. Qualitative analysis grid | 1. Statement of purpose. 2. Disclosure of authorship. 3. Disclosure of author credentials. 4. References. 5. Sources clear. 6. Email address. 7. Findability. 8. Interaction or use of interactivity. 9. Inclusion of definition. 10. Clinical features. 11. Management, with description of each treatment and how it works. |
| 79. Measurable criteria for credibility score for diabetes sites [78] | Authors | Yes | 1. Explanation of   methods   1. Validity of   methods   1. Currency of   information   1. Comprehensiveness of information 2. Accuracy of information | 1. Disclosure of authorship. 2. Disclosure of author credentials. 3. Email address. 4. References. 5. Editorial review process. 6. Disclosure of date of creation. 7. Disclosure of date of last update. 8. Inclusion of definition. 9. Epidemiology. 10. Etiology. 11. Pathogenesis. 12. Clinical features. 13. Diagnosis. 14. Management, with description of each treatment and how it works. 15. Monitoring. 16. Complications. 17. Self-management. 18. Balance between benefits and harms. 19. Description of what would happen if no treatment is used. 20. Questions to discuss with those involved in the patient’s care. 21. Costs. 22. Cases/examples of desired behavior modeled or shown. 23. Motivation. |
| 80. Multiple sclerosis evaluation instrument [79] | Authors | Yes |  | 1. Disclosure of ownership. 2. Disclosure of authorship. 3. Author is a recognized authority. 4. Pathogenesis. 5. Diagnosis. 6. Clinical features. 7. Management, with description of each treatment and how it works. 8. Complications. 9. Balance between benefits and harms. 10. Self-management. 11. Based on current research using MEDLINE searches. 12. Questions to discuss with those involved in the patient’s care. 13. Coverage of areas of uncertainty. 14. Motivation. 15. Cases/examples of desired behavior modeled or shown. 16. Available to people with disabilities or low-end technology. 17. Feedback mechanisms, response time for feedback, feature to rate the usefulness of information. 18. Email address. |
| 81. Online vaccination information quality indices [80] | Authors | No | 1. Webpage design 2. Interactivity 3. Health-related content 4. Vaccination-specific content | 1. Links provided with responsible partnering. 2. Menu (directional icons, bars, indicators, listing, indexes). 3. Easy navigation. 4. Internal search engine. 5. Type of material, cover images, illustrations, and media used to communicate. 6. Relevant graphics and images. 7. Interaction or use of interactivity. 8. Email address. 9. Disclosure of ownership. 10. Target audience clearly defined. 11. Disclosure of date of creation. 12. Disclosure of date of last update. 13. References. 14. Feedback mechanisms, response time for feedback, feature to rate the usefulness of information. 15. Advertisements distinctly labeled and separated from website content. 16. Use of a readability tool. 17. General disclaimers. 18. Inclusion of definition. 19. Management, with description of each treatment and how it works. 20. Balance between benefits and harms. |
| 82. Score sheet for evaluating Breastfeeding education materials [81] | Author | No |  | 1. Disclosure of authorship. 2. Disclosure of sponsorship. 3. Type of material, cover images, illustrations, and media used to communicate. 4. Target audience clearly defined. 5. Motivation. 6. General disclosures (educational, nonprofit, or commercial). 7. Balance between benefits and harms. 8. Relevant graphics and images. |
| 83. Website Evaluation Instrument-Female Hypoactive Sexual Desire Disorder [82] | Authors | No | 1. Demographics 2. Explanation of methods 3. Validity of methods 4. Currency of information 5. Navigability 6. Comprehensiveness 7. Accuracy of Information | 1. Disclosure of sponsorship. 2. Target audience clearly defined. 3. Use of a readability tool. 4. Disclosure of authorship. 5. Disclosure of author affiliation. 6. Disclosure of author credentials. 7. References. 8. Editorial review process. 9. Disclosure of date of last update. 10. Easy navigation. 11. Internal search engine. 12. Links provided with responsible partnering. 13. Etiology. 14. Epidemiology. 15. Diagnosis. 16. Self-management. 17. Management, with description of each treatment and how it works. 18. Balance between benefits and harms. 19. Questions to discuss with those involved in the patient’s care. 20. Clinical features. 21. Guidelines/standards of care. |
| 84. Consistency Analysis Instrument [83] | Authors | No |  | 1. Layout with appropriate illustrations adjacent to the related text; visual cueing devices such as boxes, arrows, and shading used to direct attention to key content. 2. Visual aspect. 3. Available to people with disabilities or low-end technology. 4. Feedback mechanisms, response time for feedback, feature to rate the usefulness of information. 5. Entire page loads in less than 5 seconds. 6. Easy navigation. 7. Findability. 8. Subheadings and chunking. 9. Appropriate color contrast. 10. Appropriate typography. 11. Appropriate grammar. |
| 85. Guidelines for designing user interface^e^ [84] | Authors | No | 1. Navigating the interface 2. Organizing the display 3. Interaction style 4. Getting the user’s attention 5. Golden rules of interface design | 1. Easy navigation. 2. Layout with appropriate illustrations adjacent to the related text; visual cueing devices such as boxes, arrows, and shading used to direct attention to key content. 3. Quality of visual presentation. 4. Option to download/print materials. 5. Relevant graphics and images. 6. Appropriate color contrast. 7. Type of material, cover images, illustrations, and media used to communicate. 8. Visual aspect. 9. Relevant graphics and images. 10. Cultural match: appropriate language for the region. 11. Appropriate grammar. 12. Menu (directional icons, bars, indicators, listing, indexes). 13. Feedback mechanisms, response time for feedback, feature to rate the usefulness of information. 14. Email address. 15. Appropriate typography. 16. Entire page loads in less than 5 seconds. |
| 86. Jim Kapoun's Criteria for Evaluating Web Page^b^ [85] | Author | No | 1. Accuracy 2. Authority 3. Objectivity 4. Currency 5. Coverage | 1. Email address. 2. Fax number. 3. Disclosure of authorship. 4. Disclosure of ownership. 5. General disclosures (educational, nonprofit, or commercial). 6. Disclosure of author credentials. 7. References. 8. Disclosure of physician credentials. 9. Author is a recognized authority. 10. Advertisements distinctly labeled and separated from website content. 11. Target audience clearly defined. 12. Expert consultation or personal opinion of the author. 13. Disclosure of date of creation. 14. Disclosure of date of last update. 15. Does not require other computer applications for viewing or links are provided to download needed browser plug-in. 16. Available to people with disabilities or low-end technology. 17. Relevant graphics and images. 18. Browser compatibility. |
| 87. Protocol Analysis [86] | Authors | Yes | 1. Content 2. Navigation 3. Interactivity | 1. Menu (directional icons, bars, indicators, listing, indexes). 2. Relevant graphics and images. 3. Easy navigation. 4. Feedback mechanisms, response time for feedback, feature to rate the usefulness of information. 5. Interaction or use of interactivity. |
| 88. The framework for website evaluation [87] | Authors | No | 1. Usefulness   (usability)   1. Service quality 2. Physical accessibility | 1. Easy navigation. 2. Internal search engine. 3. Layout with appropriate illustrations adjacent to the related text; visual cueing devices such as boxes, arrows, and shading used to direct attention to key content. 4. Findability. 5. Visual aspect. 6. Appropriate typography. 7. Quality of visual presentation. 8. Appropriate color contrast. 9. Type of material, cover images, illustrations, and media used to communicate. 10. Disclosure of date of creation. 11. Entire page loads in less than 5 seconds. 12. Feedback mechanisms, response time for feedback, feature to rate the usefulness of information. 13. Available to people with disabilities or low-end technology. |
| 89. Website evaluation questionnaire^a^ [88] | Authors | Yes |  | 1. Findability. 2. Links provided with responsible partnering. 3. Menu (directional icons, bars, indicators, listing, indexes). 4. Layout with appropriate illustrations adjacent to the related text; visual cueing devices such as boxes, arrows, and shading used to direct attention to key content. 5. Visual aspect. 6. Appropriate writing style. 7. Appropriate sentence construction. 8. Appropriate grammar. 9. Internal search engine. |
| 90. Web Site Quality Evaluation Instrument [89] | Authors | No | 1. Content 2. Functionality 3. Currency and stability 4. Links 5. Graphics 6. Authority 7. Coverage 8. Style | 1. Appropriate grammar. 2. References. 3. Based on current research using MEDLINE searches. 4. Target audience clearly defined. 5. General disclosures (educational, nonprofit, or commercial). 6. Cultural match: appropriate language for the region. 7. Author is a recognized authority. 8. Sources clear. 9. Easy navigation. 10. Menu (directional icons, bars, indicators, listing, indexes). 11. Subheadings and chunking. 12. Entire page loads in less than 5 seconds. 13. Disclosure of date of creation. 14. Disclosure of date of last update. 15. Date of technical maintenance. 16. Links provided with responsible partnering. 17. Relevant graphics and images. 18. Appropriate color contrast. 19. Appropriate typography. 20. Type of material, cover images, illustrations, and media used to communicate. 21. Disclosure of authorship. 22. Disclosure of author credentials. 23. Email address. 24. Fax number. 25. Disclosure of sponsorship. 26. Country. 27. Statement of purpose. 28. General disclosures (educational, nonprofit, or commercial). 29. Layout with appropriate illustrations adjacent to the related text; visual cueing devices such as boxes, arrows, and shading used to direct attention to key content. |
| 91. Website evaluation questionnaire for evaluating informational websites [90] | Authors | Yes | 1. Content 2. Navigation 3. Layout | 1. Cultural match: appropriate language for the region. 2. Appropriate writing style. 3. Appropriate sentence construction. 4. Appropriate grammar. 5. Easy navigation. 6. Menu (directional icons, bars, indicators, listing, indexes). 7. Entire page loads in less than 5 seconds. 8. Internal search engine. 9. Layout with appropriate illustrations adjacent to the related text; visual cueing devices such as boxes, arrows, and shading used to direct attention to key content. 10. Links provided with responsible partnering. |
| 92. Website Usability Evaluation Instrument (WEBUSE) [91] | Authors | Yes | 1. Content, organization, and readability 2. Navigation and links 3. User interface design 4. Performance and effectiveness | 1. Disclosure of date of last update. 2. Findability. 3. Cultural match: appropriate language for the region. 4. Subheadings and chunking. 5. Menu (directional icons, bars, indicators, listing, indexes). 6. Easy navigation. 7. Appropriate writing style. 8. Appropriate sentence construction. 9. Layout with appropriate illustrations adjacent to the related text; visual cueing devices such as boxes, arrows, and shading used to direct attention to key content. 10. Disclosure of date of creation. 11. Links provided with responsible partnering. 12. Visual aspect. 13. Quality of visual presentation. 14. Appropriate color contrast. 15. Advertisements distinctly labeled and separated from website content. 16. Entire page loads in less than 5 seconds. 17. Date of technical maintenance. 18. Option to download/print materials. |

^a^These instruments considered domains in our study as items.

^b^These instruments mentioned additional items specific for financial transactions, whether the site is free or payable.

^c^This instrument mentioned additional items specific for evaluation of telehealth websites.

^d^The original citation of these instruments could not be retrieved; the article mentioning the instrument provided items and domains.

^e^These instruments mentioned additional items specific for html coding.

NR, validation status not reported.

**References of the instruments included in our analysis:**

1. Eichner J, Dullabh P. Accessible Health Information Technology (IT) for Populations with Limited Literacy:A Guide for Developers and Purchasers of Health IT Rockville, MD2007 [October 11, 2016]. Available from: <https://healthit.ahrq.gov/sites/default/files/docs/page/LiteracyGuide_0.pdf>.

2. Price SL, Hersh WR. Filtering Web pages for quality indicators: an empirical approach to finding high quality consumer health information on the World Wide Web. Proceedings AMIA Symposium. 1999:911-5. Epub 1999/11/24. PubMed PMID: 10566493; PubMed Central PMCID: PMCPmc2232852.

3. Web Medica Acreditada. Certified Medical Web, [August 17,2016]. Available from: <http://wma.comb.es/es/wma/quees.php>.

4. Eysenbach G. Infodemiology: The epidemiology of (mis)information. The American journal of medicine. 2002;113(9):763-5. Epub 2003/01/09. PubMed PMID: 12517369.

5. BIREME / PAHO / WHO Latin American and Caribbean Center on Health Sciences Information. Criteria for the Selection of Health Information Sources Available on the Internet , [August 17,2016]. Available from: <http://bvsmodelo.bvsalud.org/download/lis/LIS-2-CriteriosSelecaoFontes-en.pdf>.

6. Narhi U, Pohjanoksa-Mantyla M, Karjalainen A, Saari JK, Wahlroos H, Airaksinen MS, et al. The DARTS tool for assessing online medicines information. Pharm World Sci. 2008;30(6):898-906. doi: <http://dx.doi.org/10.1007/s11096-008-9249-9>. PubMed PMID: 18791806.

7. Charnock D, Shepperd S, Needham G, Gann R. DISCERN: an instrument for judging the quality of written consumer health information on treatment choices. Journal of epidemiology and community health. 1999;53(2):105-11. Epub 1999/07/09. PubMed PMID: 10396471; PubMed Central PMCID: PMCPmc1756830.

8. Chumber S, Huber J, Ghezzi P. A methodology to analyze the quality of health information on the internet: the example of diabetic neuropathy. The Diabetes educator. 2015;41(1):95-105. Epub 2014/12/07. doi: 10.1177/0145721714560772. PubMed PMID: 25480397.

9. Commission of the European Communities. eEurope 2002: Quality Criteria for Health Related Websites. Journal of medical Internet research. 2002;4(3):E15. Epub 2003/01/30. doi: 10.2196/jmir.4.3.e15. PubMed PMID: 12554546; PubMed Central PMCID: PMCPmc1761945.

10. iHealthCoalition. eHealth Code of Ethics [Augsut 17, 2016]. Available from: <http://www.ihealthcoalition.org/ehealth-code-of-ethics/>.

11. European commission. Evaluation and review of the ePrivacy Directive 2016 [Octoberr 28, 2016]. Available from: <http://ec.europa.eu/justice/data-protection/article-29/documentation/opinion-recommendation/files/2016/wp240_en.pdf>.

12. Fremont P, Labrecque M, Legare F, Baillargeon L, Misson L. [Evaluation of medical web sites. Interobserver and intraobserver reliability of an evaluation tool]. Can Fam Physician. 2001;47:2270-8. PubMed PMID: 11768925; PubMed Central PMCID: PMCPMC2018466.

13. Whitten P, Holtz B, Cornacchione J, Wirth C. An evaluation of telehealth websites for design, literacy, information and content. Journal of telemedicine and telecare. 2011;17(1):31-5. Epub 2010/11/16. doi: 10.1258/jtt.2010.091208. PubMed PMID: 21075801.

14. Growth House. Rating Criteria and Excellence Awards, 2016 [August 5, 2016 ]. Available from: <http://www.growthhouse.org/award.html>.

15. Winker MA, Flanagin A, Chi-Lum B, White J, Andrews K, Kennett RL, et al. Guidelines for medical and health information sites on the internet: principles governing AMA web sites. American Medical Association. Jama. 2000;283(12):1600-6. Epub 2000/03/29. PubMed PMID: 10735398.

16. The Children's Partnership. Guidelines For Content Creation And Evaluation [August 22, 2016 ]. Available from: <http://www.childrenspartnership.org/wp-content/uploads/2016/06/The-Childrens-Partnerships-Guidelines-for-Content-Creation-and-Evaluation%E2%80%94Version-1.0_October-2003.pdf>.

17. Monsivais D, Reynolds A. Developing and evaluating patient education materials. Journal of continuing education in nursing. 2003;34(4):172-6. Epub 2003/07/31. PubMed PMID: 12887228.

18. U.S. Department of Health and Human Services Office of Disease Prevention and Health Promotion. Health literacy online: A guide to writing and designing easy-to-use health Web sites [October 28, 2016]. Available from: <https://health.gov/healthliteracyonline/2010/Web_Guide_Health_Lit_Online.pdf>.

19. Utilization Review Accreditation Commission. Health web site check-up service and accreditation program [17 August , 2016]. Available from: <https://www.urac.org/wp-content/uploads/STDGlance_HealthWebSite.pdf>.

20. Pealer LN, Dorman SM. Evaluating health-related Web sites. The Journal of school health. 1997;67(6):232-5. Epub 1997/08/01. PubMed PMID: 9285869.

21. Lewiecki EM, Rudolph LA, Kiebzak GM, Chavez JR, Thorpe BM. Assessment of osteoporosis-website quality. Osteoporosis international : a journal established as result of cooperation between the European Foundation for Osteoporosis and the National Osteoporosis Foundation of the USA. 2006;17(5):741-52. Epub 2006/02/01. doi: 10.1007/s00198-005-0042-5. PubMed PMID: 16447010.

22. Lynch KR, Schwerha DJ, Johanson GA. Development of a weighted heuristic for website evaluation for older adults. International Journal of Human-Computer Interaction. 2013;29(6):404-18.

23. Health On The Net Foundation. HONcode, [August 17, 2016]. Available from: <https://www.healthonnet.org/HONcode/Conduct.html>.

24. Biermann JS, Golladay GJ, Greenfield ML, Baker LH. Evaluation of cancer information on the Internet. Cancer. 1999;86(3):381-90. Epub 1999/08/03. PubMed PMID: 10430244.

25. American Public Health Association. Criteria for assessing the quality of health information on the Internet. American Journal of Public Health. 2001;91(3):513.

26. Stout PA, Villegas J, Kim H. Enhancing learning through use of interactive tools on health-related websites. Health education research. 2001;16(6):721-33. Epub 2002/01/10. PubMed PMID: 11780710.

27. Silberg WM, Lundberg GD, Musacchio RA. Assessing, controlling, and assuring the quality of medical information on the Internet: Caveant lector et viewor--Let the reader and viewer beware. Jama. 1997;277(15):1244-5. Epub 1997/04/16. PubMed PMID: 9103351.

28. Childs S. Developing health website quality assessment guidelines for the voluntary sector: outcomes from the Judge Project. Health information and libraries journal. 2004;21 Suppl 2:14-26. Epub 2004/08/20. doi: 10.1111/j.1740-3324.2004.00520.x. PubMed PMID: 15317572.

29. Lehigh University. Web Resource Evaluation 2016 [updated August 8,2016]. Available from: <http://library.lehigh.edu/teaching_support/information_literacy_teaching_research_skills/tutorials_research_success>.

30. Minervation validation instrument for health care web sites LIDA tool [August 13, 2016]. Available from: <http://www.minervation.com/lida-tool/>.

31. European Blind Union -The voice of blind and partially sighted people in Europe. Making information accessible for all [August 22 , 2016]. Available from: <http://www.euroblind.org/resources/guidelines/nr/88#What_is_accessible_information>.

32. Information CfIRCLaEoH. MedCIRCLE [October 1, 2016]. Available from: <http://www.medcircle.org/>.

33. NIH U.S. National Library of Medicine MedlinePlus. MedlinePlus Guide to Healthy Web Surfing [Augsut 22, 2016]. Available from: <https://medlineplus.gov/healthywebsurfing.html>.

34. Association des Centraliens. Net Scoring -criteria to assess the quality of Health Internet information, [Augsut 17, 2016]. Available from: <http://www.chu-rouen.fr/netscoring/netscoringeng.html>.

35. Tsai SL, Chai SK. Developing and validating a nursing website evaluation questionnaire. Journal of advanced nursing. 2005;49(4):406-13. Epub 2005/02/11. doi: 10.1111/j.1365-2648.2004.03304.x. PubMed PMID: 15701155.

36. Norman F. Organizing medical networked information (OMNI). Medical informatics = Medecine et informatique. 1998;23(1):43-51. Epub 1998/06/10. PubMed PMID: 9618682.

37. Clark PM, Gomez EG. Details on demand: consumers, cancer information, and the Internet. Clin J Oncol Nurs. 2001;5(1):19-24. PubMed PMID: 11899396.

38. Fraquelli M, Conte D, Camma C, Casazza G, Di Bona D, Rebulla P, et al. Quality-related variables at hepatological websites. Digestive and liver disease : official journal of the Italian Society of Gastroenterology and the Italian Association for the Study of the Liver. 2004;36(8):533-8. Epub 2004/09/01. doi: 10.1016/j.dld.2004.02.011. PubMed PMID: 15334774.

39. Shoemaker SJ, Wolf MS. The Patient Education Materials Assessment Tool (PEMAT) and User’s Guide 2013 [August 5, 2016]. Available from: <http://www.ahrq.gov/professionals/prevention-chronic-care/improve/self-mgmt/pemat/index.html>.

40. Nicoll LH. Tips, tools, and techniques. Quick and effective website evaluation. Lippincott's Case Management. 2001;6(5):220-1. PubMed PMID: 107071339. Language: English. Entry Date: 20011130. Revision Date: 20150711. Publication Type: Journal Article. Journal Subset: Double Blind Peer Reviewed.

41. The National Academy Press. For the Record Protecting Electronic Health Information: National Academies Press (US); 1997 [October 28, 2016]. Available from: <https://www.nap.edu/catalog/5595/for-the-record-protecting-electronic-health-information>.

42. Sajid MS, Iftikhar M, Monteiro RS, Miles AF, Woods WG, Baig MK. Internet information on colorectal cancer: commercialization and lack of quality control. Colorectal disease : the official journal of the Association of Coloproctology of Great Britain and Ireland. 2008;10(4):352-6. Epub 2007/07/25. doi: 10.1111/j.1463-1318.2007.01316.x. PubMed PMID: 17645570.

43. Daraz L, MacDermid JC, Wilkins S, Shaw L. Tools to Evaluate the Quality ofWeb Health Information: A Structured Review of Content and Usability. THE INTERNATIONAL JOURNAL OF TECHNOLOGY, KNOWLEDGE AND SOCIETY. 2009;5(3):127-41. PubMed PMID: ISSN 1832-3669.

44. Bohacek L, Gomez M, Fish JS. An evaluation of internet sites for burn scar management. J Burn Care Rehabil. 2003;24(4):246-51; discussion 5. PubMed PMID: 14501424.

45. Bernard A, Langille M, Hughes S, Rose C, Leddin D, Veldhuyzen van Zanten S. A systematic review of patient inflammatory bowel disease information resources on the World Wide Web. The American journal of gastroenterology. 2007;102(9):2070-7. Epub 2007/05/22. doi: 10.1111/j.1572-0241.2007.01325.x. PubMed PMID: 17511753.

46. Sandvik H. Health information and interaction on the internet: a survey of female urinary incontinence. BMJ (Clinical research ed). 1999;319(7201):29-32. Epub 1999/07/03. PubMed PMID: 10390457; PubMed Central PMCID: PMCPmc28152.

47. British Healthcare Internet Association. Quality standards for medical publishing on the web. BHIA Documents. 1996.

48. Bermudez-Tamayo C, Jimenez-Pernett J, Garcia Gutierrez JF, Azpilicueta Cengotitobengoa I, Milena Silva-Castro M, Babio G, et al. Questionnaire to evaluate health web sites according to European criteria. Atencion primaria / Sociedad Espanola de Medicina de Familia y Comunitaria. 2006;38(5):268-74. Epub 2006/10/06. PubMed PMID: 17020711.

49. Hoekstra A, Payeur B. Vaccination resources for health care providers. Journal of the American Pharmacists Association: JAPhA. 2016;56(2):161-5. doi: 10.1016/j.japh.2015.12.009. PubMed PMID: 114187689. Language: English. Entry Date: In Process. Revision Date: 20160725. Publication Type: journal article. Journal Subset: Biomedical.

50. The Quality Information Checklist QUICK [cited 2009 September 16, 2016]. Available from: <http://www.learnnc.org/lp/external/1943>.

51. Doak CC, Doak LG, Root JH. Teaching Patients with Low Literacy Skills. AJN The American Journal of Nursing. 1996;96(12):16M. PubMed PMID: 00000446-199612000-00022.

52. Jones J. Development of a self-assessment method for patients to evaluate health information on the Internet. Proceedings AMIA Symposium. 1999:540-4. Epub 1999/11/24. PubMed PMID: 10566417; PubMed Central PMCID: PMCPmc2232549.

53. SPAT Website Evaluation tool. SPAT Website Evaluation tool [August 23, 2016]. Available from: <http://www.spat.pitt.edu/>.

54. Stanford Web Credibility Research. Stanford Guidelines for Web Credibility [August 22, 2016]. Available from: <https://credibility.stanford.edu/guidelines/>.

55. Roberts L. Health information and the Internet: The 5 Cs website evaluation tool. Br J Nurs. 2010;19(5):322-5. PubMed PMID: 20335904.

56. González MP, Granollers T, Pascual A, Lorés J. Testing Website Usability in Spanish-Speaking Academia through Heuristic Evaluation and Cognitive Walkthroughs. J UCS. 2008;14(9):1513-28.

57. Nielsen J. Usability engineering: Elsevier; 1994.

58. U.S. Department of Health & Human Services. Web and Usability Guidelines [August 18, 2016]. Available from: <https://www.usability.gov/sites/default/files/documents/guidelines_book.pdf>.

59. Provost M, Koompalum D, Dong D, Martin BC. The initial development of the WebMedQual scale: domain assessment of the construct of quality of health web sites. International journal of medical informatics. 2006;75(1):42-57. Epub 2005/09/20. doi: 10.1016/j.ijmedinf.2005.07.034. PubMed PMID: 16169770.

60. Kang N, Kim J, Tack G, Hyun T. Criteria for the websites in Korean with health information on the internet. Journal of Korean Society of Medical Informatics. 1999;5(1):119-24.

61. World Wide Web Consortium (W3C), Caldwell Ben , Cooper Michael WC, Reid Loretta Guarino, Vanderheiden Gregg Web Content Accessibility Guideline 2008 [August 12, 2016]. Available from: <https://www.w3.org/TR/WCAG20/>.

62. Guardiola-Wanden-Berghe R, Gil-Perez JD, Sanz-Valero J, Wanden-Berghe C. Evaluating the quality of websites relating to diet and eating disorders. Health information and libraries journal. 2011;28(4):294-301. Epub 2011/11/05. doi: 10.1111/j.1471-1842.2011.00961.x. PubMed PMID: 22051128.

63. Consumer union Policy & action from consumer reports. Consumer Reports WebWatch Guidelines [August 22. 2017]. Available from: <http://consumersunion.org/news/consumer-reports-webwatch-guidelines/>.

64. Rolland Y, Bousquet C, Pouliquen B, Le Beux P, Fresnel A, Duvauferrier R. Radiology on Internet: advice in consulting websites and evaluating their quality. European radiology. 2000;10(5):859-66. Epub 2000/05/24. doi: 10.1007/s003300051020. PubMed PMID: 10823649.

65. Ansani NT, Vogt M, Henderson BA, McKaveney TP, Weber RJ, Smith RB, et al. Quality of arthritis information on the Internet. American journal of health-system pharmacy : AJHP : official journal of the American Society of Health-System Pharmacists. 2005;62(11):1184-9. Epub 2005/06/29. PubMed PMID: 15984050.

66. Aslani A, Pournik O, Abu-Hanna A, Eslami S. Web-site evaluation tools: a case study in reproductive health information. Studies in Health Technology & Informatics. 2014;205:895-9. PubMed PMID: 25160317.

67. Fricke M, Fallis D, Jones M, Luszko GM. Consumer health information on the Internet about carpal tunnel syndrome: indicators of accuracy. The American journal of medicine. 2005;118(2):168-74. Epub 2005/02/08. doi: 10.1016/j.amjmed.2004.04.032. PubMed PMID: 15694903.

68. Smart JM, Burling D. Radiology and the internet: a systematic review of patient information resources. Clinical radiology. 2001;56(11):867-70. Epub 2001/10/18. doi: 10.1053/crad.2001.0738. PubMed PMID: 11603887.

69. Smith D. What makes a good web site? British journal of urology. 1997;80 Suppl 3:16-9. Epub 1998/01/01. PubMed PMID: 9415079.

70. Seomun GA, Lee SJ, Chang SO, Lee SJ. An evaluation study of dementia information providing websites in Korea. Taehan Kanho Hakhoe chi. 2005;35(3):631-40. Epub 2005/07/20. PubMed PMID: 16027515.

71. Schloman BF. Whom do you trust? Evaluating internet health resources. Online J Issues Nurs. 1999;4(1).

72. Wyatt JC. Commentary: measuring quality and impact of the World Wide Web. BMJ (Clinical research ed). 1997;314(7098):1879-81. Epub 1997/06/28. PubMed PMID: 9224133; PubMed Central PMCID: PMCPmc2127007.

73. Whitten P, Nazione S, Lauckner C. Tools for assessing the quality and accessibility of online health information: initial testing among breast cancer websites. Informatics for health & social care. 2013;38(4):366-81. Epub 2013/08/21. doi: 10.3109/17538157.2013.812644. PubMed PMID: 23957628.

74. Bath PA, Bouchier H. Development and application of a tool designed to evaluate web sites providing information on Alzheimer's disease. Journal of Information Science. 2003;29(4):279-97. doi: 10.1177/01655515030294005. PubMed PMID: WOS:000186515800005.

75. Barnes C, Harvey R, Wilde A, Hadzi-Pavlovic D, Wilhelm K, Mitchell PB. Review of the quality of information on bipolar disorder on the Internet. Australian & New Zealand Journal of Psychiatry. 2009;43(10):934-45. PubMed PMID: 105328425. Language: English. Entry Date: 20091204. Revision Date: 20150711. Publication Type: Journal Article.

76. Fung KM, Tihan T. Internet and World Wide Web-based tools for neuropathology practice and education. Brain pathology (Zurich, Switzerland). 2009;19(2):323-31. Epub 2009/03/18. doi: 10.1111/j.1750-3639.2009.00263.x. PubMed PMID: 19290999.

77. Givron P, Coudeyre E, Lopez S, Mares P, Herisson C, Pelissier J. Quality assessment of information about female urinary incontinence from French speaking websites. Annales de readaptation et de medecine physique : revue scientifique de la Societe francaise de reeducation fonctionnelle de readaptation et de medecine physique. 2004;47(5):217-23; discussion 24. Epub 2004/06/09. doi: 10.1016/j.annrmp.2004.03.001. PubMed PMID: 15183259.

78. Seidman JJ, Steinwachs D, Rubin HR. Design and testing of a tool for evaluating the quality of diabetes consumer-information Web sites. Journal of medical Internet research. 2003;5(4):e30. Epub 2004/01/10. doi: 10.2196/jmir.5.4.e30. PubMed PMID: 14713658; PubMed Central PMCID: PMCPmc1550576.

79. Harland J, Bath P. Assessing the quality of websites providing information on multiple sclerosis: evaluating tools and comparing sites. Health informatics journal. 2007;13(3):207-21. Epub 2007/08/23. doi: 10.1177/1460458207079837. PubMed PMID: 17711882.

80. Sak G, Diviani N, Allam A, Schulz PJ. Comparing the quality of pro- and anti-vaccination online information: a content analysis of vaccination-related webpages. BMC Public Health. 2016;16:38. doi: <http://dx.doi.org/10.1186/s12889-016-2722-9>. PubMed PMID: 26769342; PubMed Central PMCID: PMCPMC4714533.

81. Smith LJ. A score sheet for evaluating breastfeeding educational materials. Journal of human lactation : official journal of International Lactation Consultant Association. 1995;11(4):307-11. Epub 1995/12/01. PubMed PMID: 8634107.

82. Touchet BK, Warnock JK, Yates WR, Wilkins KM. Evaluating the quality of websites offering information on female hypoactive sexual desire disorder. Journal of sex & marital therapy. 2007;33(4):329-42. Epub 2007/06/02. doi: 10.1080/00926230701385555. PubMed PMID: 17541851.

83. Steinau S, Diaz O, Rodriguez JJ, Ibanez F. A tool for assessing the consistency of websites. Piattini MG, Filipe J, Braz J, editors. Dordrecht: Springer; 2002. 227-34 p.

84. Shneiderman B. Designing the user interface: strategies for effective human-computer interaction: Pearson Education India; 2010.

85. Kapoun J, Cornell University Library. Teaching undergrads WEB evaluation: A guide for library instruction. : Cornell University Press; 1998 [Augsut 22, 2016]. Available from: <http://guides.library.cornell.edu/c.php?g=32334&p=203768&preview=ad0bac0490cf7ab0653096fe3b4a0fee>.

86. Benbunan-Fich R. Using protocol analysis to evaluate the usability of a commercial web site. Information & management. 2001;39(2):151-63.

87. Qi S, Ip C, Leung R, Law R, editors. A new framework on website evaluation. E-Business and E-Government (ICEE), 2010 International Conference on; 2010: IEEE.

88. Elling S, Lentz L, de Jong M, van den Bergh H. Measuring the quality of governmental websites in a controlled versus an online setting with the ‘Website Evaluation Questionnaire’. Government Information Quarterly. 2012;29(3):383-93. doi: <http://dx.doi.org/10.1016/j.giq.2011.11.004>.

89. McInerney CR, Bird NJ. Assessing website quality in context: Retrieving information about genetically modified food on the web. Information Research: An International Electronic Journal. 2005;10(2):n2.

90. Elling S, Lentz L, De Jong M, editors. Website evaluation questionnaire: development of a research-based tool for evaluating informational websites. International Conference on Electronic Government; 2007: Springer.

91. Chiew TK, Salim SS. Webuse: Website usability evaluation tool. Malaysian Journal of Computer Science. 2003;16(1):47-57.

92. Jadad AR, Gagliardi A. Rating health information on the Internet: navigating to knowledge or to Babel? Jama. 1998;279(8):611-4. Epub 1998/03/05. PubMed PMID: 9486757.

93. Gagliardi A, Jadad AR. Examination of instruments used to rate quality of health information on the internet: chronicle of a voyage with an unclear destination. BMJ (Clinical research ed). 2002;324(7337):569-73. Epub 2002/03/09. PubMed PMID: 11884320; PubMed Central PMCID: PMCPmc78993.

94. Kim P, Eng TR, Deering MJ, Maxfield A. Published criteria for evaluating health related web sites: review. BMJ (Clinical research ed). 1999;318(7184):647-9. Epub 1999/03/05. PubMed PMID: 10066209; PubMed Central PMCID: PMCPmc27772.

95. Eysenbach G, Powell J, Kuss O, Sa ER. Empirical studies assessing the quality of health information for consumers on the world wide web: a systematic review. Jama. 2002;287(20):2691-700. Epub 2002/05/22. PubMed PMID: 12020305.

96. Sagaram S, Walji M, Meric-Bernstam F, Johnson C, Bernstam E. Inter-observer agreement for quality measures applied to online health information. Studies in health technology and informatics. 2004;107(Pt 2):1308-12. PubMed PMID: 15361026.

97. Bernstam EV, Shelton DM, Walji M, Meric-Bernstam F. Instruments to assess the quality of health information on the World Wide Web: what can our patients actually use? International journal of medical informatics. 2005;74(1):13-9. Epub 2005/01/01. doi: 10.1016/j.ijmedinf.2004.10.001. PubMed PMID: 15626632.

98. Zhang Y, Sun YL, Xie B. Quality of health information for consumers on the web: A systematic review of indicators, criteria, tools, and evaluation results. J Assoc Inf Sci Tech. 2015;66(10):2071-84. doi: 10.1002/asi.23311. PubMed PMID: WOS:000361184500008.
