# Supplementary material for: A comprehensive scoping review to identify standards for the development of health information resources on the internet
Source: PLoS One. 2019 Jun 20;14(6):e0218342. doi: 10.1371/journal.pone.0218342 (PMC6586310; doi:10.1371/journal.pone.0218342)
Supplement: S3 Table — (DOCX) [file pone.0218342.s004.docx]

**S3 Table:** Summary of previous systematic reviews evaluating the rating instruments used for evaluation of health-related information on the internet.

| **Author** | **Year** | **Number of instruments evaluated** | **Main conclusion** | **Development of a new instrument** | **Categories proposed per evaluation** |
| --- | --- | --- | --- | --- | --- |
| Jadad [92] | 1998 | 14 | A large number of incompletely developed instruments are available to evaluate health information on the internet | No | 1. Authorship 2. Attribution 3. Disclosure |
| Gagliardi [93] | 2002 | 5 | An update to the Jadad et al review; identified five new rating instruments | No | 1. Authorship 2. Attribution 3. Disclosure |
| Kim [94] | 1999 | 29 | An evaluation instrument that integrated simple consensus criteria that need to be developed and validated | Yes | 1. Content (quality, reliability, accuracy, scope, depth) 2. Design and aesthetics (layout, interactivity, presentation, appeal, graphics, use of media) 3. Disclosure of authors, sponsors, developers (identification of purpose, nature of organization, sources of support, authorship, origin) 4. Currency of information (includes frequency of update, freshness, maintenance of site) 5. Authority of source (reputation of source, credibility, trustworthiness) 6. Ease of use (usability, navigability, functionality) 7. Accessibility and availability (ease of access, fee for access, stability) 8. Links (quality of links, links to other sources) 9. Attribution and documentation (presentation of clear references, balanced evidence) 10. Intended audience (nature of intended users, appropriateness for intended users) 11. Contact addresses or feedback mechanism (availability of contact information, contact address) 12. User support (availability of support, documentation for users) 13. Miscellaneous (criteria that lacked specificity or were unique) |
| Eysenbach [95, 96] | 2002 | 86 | Operational definitions were created for the quality criteria that evaluate health information on the internet | Yes | 1. Accuracy 2. Completeness/comprehensiveness/coverage/scope 3. Technical elements   Disclosure of authorship  Disclosure of ownership  Source clear  Disclosure of sponsorship  Disclosure of advertising  Statement of purpose  General disclosure  Date of creation disclosed  Date of last update disclosed  Date of creation or update disclosed  Authors’ credentials disclosed  Credentials of physicians disclosed  Authors’ affiliations disclosed  Easy navigation  Internal search engine present  Links provided  References provided  Balanced evidence  Writing style appropriate  Feedback mechanism provided  Fax number provided  Email address provided  General disclaimer provided  Copyright notice  Editorial review process  Hierarchy of evidence clear   1. Readability 2. Design and aesthetics 3. Accessibility 4. Usability |
| Bernstam [97] | 2005 | 21 | Evaluation of instruments that can allow information consumers to appraise the quality of websites providing their health information; authors considered only instruments with 10 or fewer elements | No | 1. Date of creation or update disclosed (currency) 2. Disclosure of physician’s credentials 3. Editorial review process 4. References provided 5. Disclosure of ownership 6. Sources clear 7. Feedback mechanism (website contact information) 8. Copyright notice 9. Disclosure of author’s credentials 10. Disclosure of authorship 11. Statement of purpose 12. Disclosure of advertising 13. Links provided 14. Disclosure of sponsorship 15. Disclaimer (e.g., cannot substitute for physician’s care) 16. Disclosure of privacy policies 17. Payment policy/procedures for secure transactions 18. Graphics and multimedia present 19. Discloses whether the site charges a fee 20. Readability |
| Zhang [98] | 2015 | 155 | Many pre-existing instruments were used for evaluation of the formality of the content, but only a few of them were used in several studies and their validity remained questionable; proprietary instruments (based on textbooks and medical guidelines) were used for evaluation of the quality of the content | No | 1. Content   Substance (accuracy, completeness)  Formality  Currency (publication date, copyright date, date of last update, date of next update, response to current events)  Credibility (authorship, disclosure, attribution)  Readability (facilitated by automatic tools)   1. Design   Accessibility (operational, clearly presents contact information and registration and accession fees, accessible for disabled people, offers other languages, technical support available, reasonable load time, text-only option, design consistency, no distracting graphics or text displays, appropriate fonts)  Aesthetics (site layout, appropriate images, use of headings, color schema)  Navigability (navigation structure, site map, easy to return to homepage, always tells current location, consistency of menu systems)  Interactivity (information exchange, internal search engine, multimedia capability, frequently asked questions section, personalization, social media expansion)  Privacy and data protection (policy on data collection and use of personal data, can use site anonymously)  Cultural sensitivity (logic, language, and experience of the content match the intended audiences and present cultural images and examples in positive and realistic ways) |

**References of previous systematic reviews evaluating the rating instruments used for evaluation of health-related information on the internet:**

1. Jadad AR, Gagliardi A. Rating health information on the Internet: navigating to knowledge or to Babel? Jama. 1998;279(8):611-4.

2. Gagliardi A, Jadad AR. Examination of instruments used to rate quality of health information on the internet: chronicle of a voyage with an unclear destination. BMJ (Clinical research ed). 2002;324(7337):569-73.

3. Kim P, Eng TR, Deering MJ, Maxfield A. Published criteria for evaluating health related web sites: review. BMJ (Clinical research ed). 1999;318(7184):647-9.

4. Eysenbach G, Powell J, Kuss O, Sa ER. Empirical studies assessing the quality of health information for consumers on the world wide web: a systematic review. Jama. 2002;287(20):2691-700.

5. Sagaram S, Walji M, Meric-Bernstam F, Johnson C, Bernstam E. Inter-observer agreement for quality measures applied to online health information. Studies in health technology and informatics. 2004;107(Pt 2):1308-12.

6. Bernstam EV, Shelton DM, Walji M, Meric-Bernstam F. Instruments to assess the quality of health information on the World Wide Web: what can our patients actually use? International journal of medical informatics. 2005;74(1):13-9.

7. Zhang Y, Sun YL, Xie B. Quality of health information for consumers on the web: A systematic review of indicators, criteria, tools, and evaluation results. J Assoc Inf Sci Tech. 2015;66(10):2071-84.
